# 3D-CNN detection of systemic symptoms induced by different *Potexvirus* infections in four *Nicotiana benthamiana* genotypes using leaf hyperspectral imaging

**DOI:** 10.1186/s13007-025-01337-0

**Published:** 2025-02-10

**Authors:** Rizos-Theodoros Chadoulis, Ioannis Livieratos, Ioannis Manakos, Theodore Spanos, Zeinab Marouni, Christos Kalogeropoulos, Constantine Kotropoulos

**Affiliations:** 1 Department of Informatics, Aristotle University of Thessaloniki, University Campus, 54124 Thessaloniki, C. Macedonia Greece; 2 Information Technologies Institute, Centre for Research and Technology Hellas, 6th km Charilaou-Thermi Rd, Thermi, 57001 Thessaloniki, C. Macedonia Greece; 3 Department of Sustainable Agriculture, Mediterranean Agronomic Institute of Chania, Alsylio Agrokepio, 73100 Chania, Crete Greece

**Keywords:** Artificial intelligence, Convolutional neural networks, Dimensionality reduction, Hyperspectral imaging, Early plant disease detection

## Abstract

**Purpose:**

Hyperspectral imaging combined with machine learning offers a promising, cost-effective alternative to invasive chemical analysis for early plant disease detection. In this study, the use of 3D Convolutional Neural Networks (3D-CNNs) was explored to detect presymptomatic viral infections in the model plant *Nicotiana benthamiana L.* and assess the generalization of these models across different plant genotypes.

**Methods:**

Four genotypes of *Nicotiana benthamiana L.* (wild-type, *DCL2/4*, *AGO2*, and *NahG*) were inoculated with different *potexviruses* (PepMV mild or severe strain, PVX, BaMV). Viral infection was verified via northern blot analysis at 5 and 10 days post inoculation (DPI). Hyperspectral images were captured over 10 days following inoculation, focusing on the top 3 leaves where symptoms typically appear. The dataset was carefully processed to remove errors, and raster masks were generated to isolate only the leaf pixels. The Extremely Randomized Trees algorithm was used for Effective Wavelength selection, and a novel 3D-CNN architecture was developed to classify $16 \times 16 \times 16$ nonoverlapping cubes extracted from the unmasked leaf surfaces. The aim was to classify each cube into healthy or diseased for each of the four viruses at different time points.

**Results:**

Accuracies of $0.78$–$0.87$ were achieved for *AGO2* mutants at the cube level, and overall plant-level accuracies of $0.68$–$0.89$. The model’s generalization capabilities were tested across other genotypes, yielding accuracies of up to $0.75$ for *DCL2/4*, $0.83$ for *NahG*, and $0.78$ for the wild-type. The timing of disease detection was also assessed, finding that accuracies approached 0.8 as early as $6$–$8$ DPI depending on the virus. The results were validated against northern blot analyses and benchmarked against another state-of-the-art methodology for *Nicotiana benthamiana* viral infections, achieving superior overall classification accuracies.

**Conclusion:**

The proposed patch-based method demonstrated key advantages: (a) exploiting both spectral and textural information, (b) deriving a large training dataset from few hyperspectral images, (c) providing localized classification explainability within leaf regions, and (d) achieving high accuracy for early detection of viral infections.

## Background

Over $80\%$ of the human diet is plant-based [1], and crop losses due to biotic agents represent a serious threat to food security with an economic impact globally between 20–$40\%$ [2]. Plant viruses and viroids, the second most economically significant class of plant pathogens after fungi [3], account for approximately $\$30$ billion of crop losses annually [4–6]. Specifically, the RNA virus *Tomato spotted wilt virus* is responsible for more than $\$1$ billion in losses per [7], while two DNA viruses, *Tomato yellow leaf curl virus* and *African cassava mosaic virus*, combined may cause $\$1.9$–2.7 billion losses annually [8]. Farm practices aiming to restrict virus infections *via* the limitation of vector populations (aphids, whiteflies) typically involve intensive applications of agrochemicals, resulting in adverse effects on the agroecosystem. Early diagnosis and containment of viral pathogens can play an important role in minimizing economic losses and can benefit the agroecosystem while providing environmental protection [9]. To this end, reliable, sensitive, and cost-effective plant disease detection tools are needed to detect novel viruses that are increasingly emerging in temperate regions as a result of climate change and intensive global trade in the early stages of infection.

Traditionally, invasive methods have been employed for plant disease detection [10]. They predominantly consist of serological or/and molecular interactions that specifically identify viral nucleic acids or proteins present in infected tissues [11]. However, invasive methods may exhibit inconsistency or insensitivity issues due to their dependence on host-pathogen interaction and/or low virus titers in plant tissues [12]. Typically, they are time-consuming and require highly qualified personnel. During the last decades, the rise in computing power, the advances in sensor technologies, and the boom in Artificial Intelligence (the AI summer [13]) have facilitated the development of indirect (non-invasive) methods for identifying plant diseases. These methods rely on the acquisition of the reflectance at various wavelengths and its correlation with physiochemical plant phenomena (features) that are exploited by AI techniques to estimate quantitative characteristics of each feature *via* various parameters, such as morphological or temperature change. The most popular non-invasive techniques are fluorescence spectroscopy, visible/near-infrared spectroscopy, fluorescence imaging, and hyperspectral imaging [14]. This paper examines hyperspectral imaging.

Hyperspectral imaging has been proven to be valuable in the extraction of plant status information and the detection of quality traits in cultivations [15]. A wide range of approaches for early (presymptomatic) plant disease detection using hyperspectral imagery have been described. Hyperspectral imaging is usually combined with Machine Learning or Deep Learning techniques for feature selection/extraction and classification. Some research efforts involve taking measurements under field conditions using portable instruments, e.g., handheld spectroradiometers [16] or portable hyperspectral imaging systems [17], while others take place in a controlled lab environment [18]. When working in the lab environment, researchers often inoculate plants with one or more viral strains and monitor the effect on the surface reflectance of the plants’ leaves over a period following the inoculation. This period varies from a few days to months, according to how rapidly each plant species/infection develops. For tobacco plants, most studies focus on the first eight days post inoculation (DPI) [19–21], while only one study used a 20 DPI period [22].

Feature selection and/or extraction is ubiquitous in the literature. Acquiring hyperspectral images with that many bands requires expensive equipment that is accessible only to large landowners or research institutions. Thus, achieving dimensionality reduction while sustaining high classification accuracy is necessary to eradicate the consequences of the Hughes phenomenon [23] and lift the cost barrier, thus enabling the wider adoption of plant disease detection techniques. Towards that end, a wide range of methods, including the successive projections algorithm (SPA) [19–21], boosted regression tree (BRT) [19], genetic algorithm (GA), [19] and the grey level co-occurrence matrix [24] have already been employed for feature selection and extraction. In the context of this paper, the Extremely Randomized Trees (ERT) [25] algorithm was employed for feature selection because, compared to other frequently used feature selection algorithms (e.g., SPA, GA, BRT), it achieves a favorable trade-off among computational speed, robustness against overfitting, and ability to handle large-dimensional feature spaces, making it well-suited for hyperspectral data. ERT is an ensemble learning method that builds multiple decision trees akin to Random Forests (RF). However, it is distinctive due to its extreme level of randomization during tree construction: rather than searching for the best split among a subset of features, ERT randomly selects one feature and a random threshold for each node split, ignoring split quality. This randomization can reduce overfitting and often yields faster training compared to traditional decision trees or RF, making ERT a powerful tool for various classification and regression tasks.

A wide variety of machine-learning algorithms have been employed for plant viral disease detection. In 2014, Krezhova et al. [22] utilized hierarchical clustering to detect and assess the development of Tomato spotted wilt virus (TSWV) infection in tobacco plants. Many early research efforts resort to Support Vector Machines (SVMs). In 2017, Moghadam et al. [26] trained SVM classifiers on three types of features: vegetation indices, features based on probabilistic topic modeling, and the full spectrum. In 2021, Gui et al. [27] employed SVMs for the early detection of *Soybean mosaic virus* (SMV), and in 2022, Peng et al. [28] proposed using SVM as the classifier, along with various information fusion schemes.

The advent of deep learning has reignited research interest in plant viral disease detection, leading the scientific community to pivot from traditional approaches like Support Vector Machines (SVMs) to exploring innovative deep learning architectures. In 2016, Zhu et al. [21] employed Back Propagation Neural Networks (BPNNs) and Extreme Learning Machines (ELMs) to discriminate between mock-inoculated tobacco leaves and diseased leaves (mock-inoculated, 2 DPI, 4 DPI, 6 DPI), achieving classification accuracies of up to $95\%$. Convolutional Neural Networks (CNNs) have also proven valuable both for feature extraction and classification tasks. CNNs were originally proposed as a way to tackle computer vision problems [29, 30]. In this study, a 3D-CNN architecture, a generalization of CNNs in which the kernel moves in three directions, was used. 3D-CNNs were chosen because they can capture both spatial and spectral features. Convolutional networks have already been tested for related tasks, such as the detection of fruit quality [31]. 3D-CNNs have also been employed in plant disease detection. In particular, Sawyer et al. [18] employed 3D-CNNs to identify and distinguish leaves from Grapevine leafroll-associated virus (GLRaV) infected vines, Grapevine red blotch-infected virus (GRBV) infected vines, and vines co-infected with both viruses using spatiospectral information in the visible domain (510–710 nm). In 2021, Gui et al. [27] employed a combined CNN and SVM (CNN-SVM) method for the early detection of SMV, while Nguyen et al. [17] incorporated 2D- and 3D-CNNs for feature extraction in their experimental workflow.

In this study, four genotypes of a common plant virology indicator species (*Nicotiana benthamiana* L.), with different sensitivities to plant viruses, were inoculated with four members of the genus *Potexvirus* in order to test the capabilities of 3D-CNNs for plant disease detection. Employing a novel patch-wise approach, $16 \times 16 \times 61$ patches were extracted from hyperspectral images in the Visible/Near Infrared (Vis/NIR) spectral region (380–1023 nm) and reduced to $16 \times 16 \times 16$ using the ERT algorithm for feature selection. These patches served as input for the 3D-CNN models, which were then applied to detect viral diseases across different genotypes, achieving accuracies up to 0.8893. The models demonstrated notable generalization across genotypes, with variable accuracies that indicate the robustness of disease detection. A summary of the most recent research efforts focusing on viral disease detection using hyperspectral imaging can be found in Table 1. Our study is also included in the table for comparison purposes.

**Table 1:** Literature summary for viral disease detection using hyperspectral images plant leaves

Refs.	Year	Plant	Viruses	Context	Sampling	Classifier
Our study	2024	*Nicotiana benthamiana* (WT, *DCL2/4*, *NahG*, *AGO2*)	PepMV-SP13, PepMV-PCH 06/104, PVX, BaMV	Lab	0, 2, 4, 6, 8, 10 DPI	3D-CNN
[16]	2023	Winegrape cultivar	GLRaV-1, GLRaV-3, GLRaV-4, GVA	Field	6 timepoints in season	PLS-DA
[18]	2023	Grapevines	GLRaV, GRBV	Lab	2 timepoints in season	3D-CNN, RF
[28]	2022	Manihot esculenta	CBSV	Lab	7, 28, 52, 87 DPI	SVM
[27]	2021	Soybean	SMV	Lab	7, 15 DPI	CNN-SVM
[17]	2021	Grapevines	GVCV	Field	4 timepoints	RF, SVM, CNN
[32]	2019	Sweet pepper	TSWV	Lab	5, 7, 13 DPI	GAN
[19]	2019	*Nicotiana benthamiana*	TSWV	Lab	1, 2, 3, 4, 5, 6, 7, 8 DPI	CART, BRT, SVM, RF
[24]	2018	Tomato	TYLCV	Lab	25 DPI	GLCM
[20]	2017	*Nicotiana tabacum* L.	TMV	Lab	2, 4, 6 DPI	PLS-DA, RF, SVM, BPNN, ELM, LS-SVM
[26]	2017	*Capsicum annuum*	TSWV	Lab	3, 5, 7, 12, 21 DPI	SVM
[21]	2016	*Nicotiana tabacum* L.	TMV	Lab	2, 4, 6 DPI	BPNN, ELM
[22]	2014	*Nicotiana tabacum* L.	TSWV	Lab	14, 20 DPI	Hierarchical Clustering

Classifier names: Partial Least-Squares Discriminant Analysis (PLS-DA), Random Forest (RF), Generative Adversarial Network (GAN), Classification And Regression Tree (CART), Least Squares Support Vector Machines (LS-SVM), Back Propagation Neural Network (BPNN). Viruses: Pepino mosaic virus (PepMV), Bamboo mosaic virus (BaMV), Potato virus X (PVX), Grapevine leafroll-associated virus (GLRaV), Grapevine red blotch virus (GRBV), Cassava brown streak virus (CBSV), Soybean mosaic virus (SMV), Grapevine vein clearing virus (GVCV), Tomato spotted wilt virus (TSWV), Tomato yellow leaf curl virus (TYLCV) and Tobacco mosaic virus (TMV)

The novelty of this work lies in the following aspects:

- Most works in the literature focus on a single pathogen, and to our knowledge, there is no previous work studying the generalization capability of a computational method for plant disease detection across different genotypes of the same plant species. Our study aspires to fill this gap by employing four viruses and studying the generalization capability of our approach across four genotypes.
- A comprehensive framework for systematically identifying and removing errors and inconsistencies within the dataset without compromising the integrity of the method is proposed. This is an aspect that is usually ignored or just omitted but is, in fact, quite essential, especially in real-world situations.
- The combination of ERT for feature selection and 3D-CNN for classification is demonstrated to be more efficient than SPA for feature selection and XGBoost for classification, as can be seen in the “Benchmarking against related work” section.
- The majority of previous related studies employed a pixel-based approach that involved assigning a label to each individual pixel in an image based on its characteristics. This paper proposes a patch-based approach that involves dividing the image into smaller patches or sub-images and classifying each patch as a whole. In this way, the proposed method exploits both the spatial and the spectral domain.
- The proposed method places emphasis on early plant disease detection. Accuracies higher than 0.75 were achieved as early as 4 DPI, and the model performed particularly well when tested on hitherto unseen mutants.
- The proposed methodology outperforms similar approaches for viral disease detection when benchmarked on our dataset, and, to our knowledge, this paper is the first to use the ERT algorithm for wavelength selection in the field of plant disease detection.

## Methods

### Experimental set-up, plant material and virus inoculum

In plants, salicylic acid (SA) plays an essential role in a broad-spectrum plant innate immunity, acting as a signaling molecule following infection with biotrophic and hemibiotrophic phytopathogens. SA biosynthesis is essential for the initiation of local and systemic acquired resistance [33]. NahG transgenic *N. benthamiana* plants [34] that have reduced endogenous SA levels due to their expression of a bacterial NahG gene (salicyclic acid hydroxylase) were used in this study (kindly provided by J. Jones; Sainsbury Lab, UK). RNA silencing is an essential sequence-specific defense mechanism that protects plants from pathogen infection and also controls the regulation of gene expression [35]. The *DCL2/4* and *AGO2* genes are two critical components of the plant RNA silencing pathway. CRISPR/Cas9-generated *AGO2* [36] and DCL2/4-suppressed [37] mutant *N. benthamiana* seeds were a kind donation from K. Fatyol (Agricultural Biotechnology Institute, Godollo, 2100, Hungary) and K. Kalantidis’ (University of Crete) lab, respectively.

**Fig. 1:**
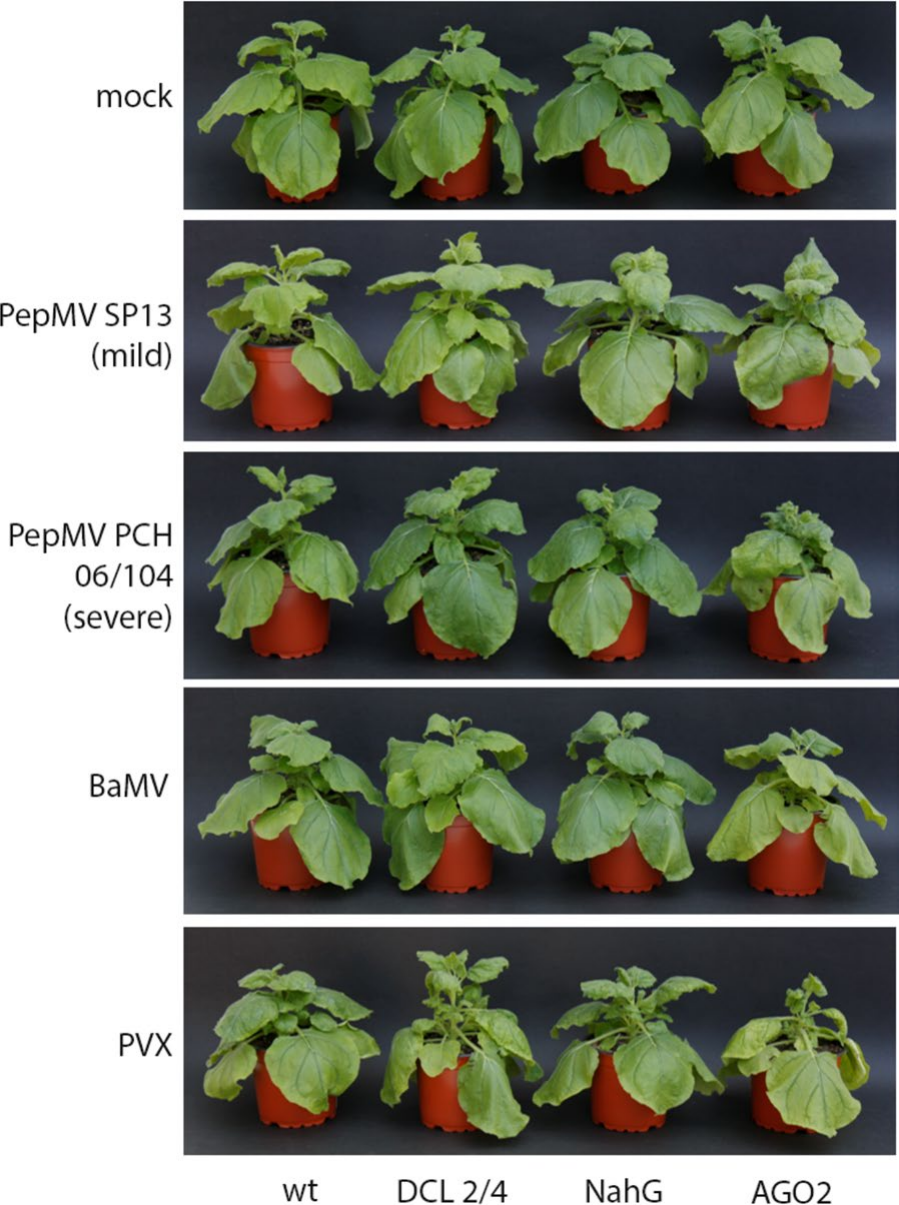
Virus-induced symptomatology induced 10 DPI following individual mechanical inoculation of four *N. benthamiana* genotypes (WT, DCL2/4, NahG, & AGO2) with four *Potexvirus*es [Pepino mosaic virus (PepMV-SP13; mild isolate, PepMV-PCH 06/104; severe isolate), Bamboo mosaic virus (BaMV) and Potato virus X (PVX)]. The inoculum consisted in all cases of 1 μgr of purified virion preparations diluted in 0.1 M Tris-borate buffer (mock). Three *N. benthamiana* plants (replications) were used in each treatment for leaf hyperspectral analysis

In this study, four genotypes of *N. benthamiana*, a plant species susceptible to a large number of viruses were mechanically inoculated with one of four members of the genus *Potexvirus*, as shown in Fig. 1. Virus-induced symptomatology was monitored for a period of 10 days ($11/09/2020$–$21/09/2020$) following infection of *N. benthamiana* wild-type (WT), *DCL2/4*, *NahG* and *AGO2* plants with Pepino mosaic virus (PepMV-SP13; mild isolate, PepMV PCH 06/104; severe isolate), Bamboo mosaic virus (BaMV) and Potato virus X (PVX). The inoculum consisted in all cases of 1 μgr of purified virion preparations suspended in 0.1*M* Tris-borate buffer. Four *N. benthamiana* plants were used for each treatment, one of which was subjected to tissue-disrupting laboratory analysis to verify infection.

### RNA extraction and Northern blot analysis

For the assessment of viral infection, systemic (two upper leaves) samples (100 mg) were collected at 5 and 10 DPI from one inoculated plant from each treatment. The leaves were ground in liquid nitrogen, and total RNA was extracted with $\text {TRIzol}^{TM}$ Reagent (Invitrogen) according to the manufacturer’s instructions. Total RNA concentration and quality were assessed using a $\text {NanoPhotometer}^{TM}$ Pearl (Implen). The integrity of the total RNA was checked by resolution (1 to 5 μg) in $1\%$ agarose/0.22 M formaldehyde agarose gel electrophoresis and transfer onto Hybond-N membranes (GE HealthCare). Northern blots were hybridized with a digoxin (DIG)-labeled riboprobe corresponding to the (–) sense of each viral coat protein (CP) gene (PepMV SP-13, PepMV-PCH 06/104, PVX, and BaMV), created by T7 in vitro transcription (Promega) using plasmid vectors harboring the CP genes.

### Measurements

A staring hyperspectral imaging system (Muses9 HS 1700) with spectral coverage from 370 to 1700 nm was utilized [38]. This imaging system made use of a two-branch light source with emission covering the entire camera’s spectral range. The wavelength step could be adjusted by the user, and was set to 10 nm. The imaging system was accompanied by a manufacturer’s software that was installed in a laptop with moderate resources (Intel i7-8550U 1.89 GHz with four cores and 16 Gb RAM). The software controls the functionality of the instrument and guides the user in avoiding the capture of saturated or damaged samples.

For the purpose of this study, we focused primarily on the spectral range up to 1000 nm. This choice was made because the region below 1000 nm is particularly rich in information related to physiological and biochemical properties of plant tissues. Visible and near-infrared (NIR) wavelengths up to 1000 nm are commonly used to assess key plant traits such as chlorophyll content, water status, and overall plant health. Given the specific focus on systemic symptoms in *Nicotiana benthamiana*, this spectral region was deemed sufficient to capture the relevant physiological changes induced by *Potexvirus* infections.

The measurements were collected in a lab environment, in the facilities of the Plant Virology and Microbiology Laboratory (Mediterranean Agronomic Institute of Chania). During the measurements, all lab windows were covered with opaque sheets to prevent the ingress of light from external sources. The camera was mounted on a frame (mounting plate) to which the light sources were attached on specialized arms. The spectrophotometer (camera and light sources) was placed on a tripod and connected to the laptop via an HDMI cable. The manufacturer’s calibration files for indoor use were employed. The measurements were exported in JPEG format.

### The structure of the dataset

Sixty *N. benthamiana* plants of 4 different genotypes: wild type (WT), DCL2/4 [37], *NahG*, and *AGO2* [36] were used. Of these plants, twelve were mock-inoculated, and the forty-eight were infected with one of four different *Potexvirus*es (PepMV SP13, PepMV-PCH 06/104, PVX, and BaMV; Table 2).

**Table 2:** A summary of the genotype and the health condition of each of the 60 plants used in the experiments, organized in groups of the same genotype and health condition

Plants	Genotype	Condition	Plants	Genotype	Treatment
1,2,3	*WT*	Mock-inoculated	31,32,33	*NahG*	PCH 06/104
4,5,6	*DCL2/4*	Mock-inoculated	34,35,36	*AGO2*	PCH 06/104
7,8,9	*NahG*	Mock-inoculated	37,38,39	*WT*	BaMV
10,11,12	*AGO2*	Mock-inoculated	40,41,42	*DCL2/4*	BaMV
13,14,15	*WT*	PepMV SP13	43,44,45	*NahG*	BaMV
16,17,18	*DCL2/4*	PepMV SP13	46,47,48	*AGO2*	BaMV
19,20,21	*NahG*	PepMV SP13	49,50,51	*WT*	PVX
22,23,24	*AGO2*	PepMV SP13	52,53,54	*DCL2/4*	PVX
25,26,27	*WT*	PCH 06/104	55,56,57	*NahG*	PVX
28,29,30	*DCL2/4*	PCH 06/104	58,59,60	*AGO2*	PVX

**Fig. 2:**
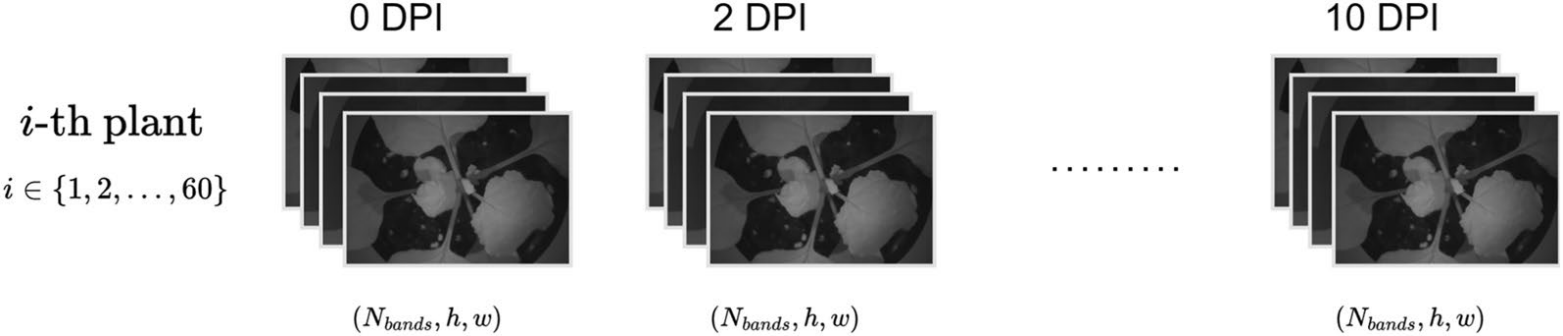
The structure of the dataset, where *h* and *w* are the spatial dimensions of the hyperspectral images in pixels, $N_{\text {bands}}$ is the number of the spectral bands and DPI refers to days post inoculation

Hyperspectral images were taken every 2 days and throughout a period of 10 days (11/9/2020–21/9/2020) for each of the 60 plants. Technically, each image is a three-dimensional data array (hypercube) containing two spatial dimensions plus an additional spectral dimension. The structure of the dataset is shown in Fig. 2.

### Experimental framework

**Fig. 3:**
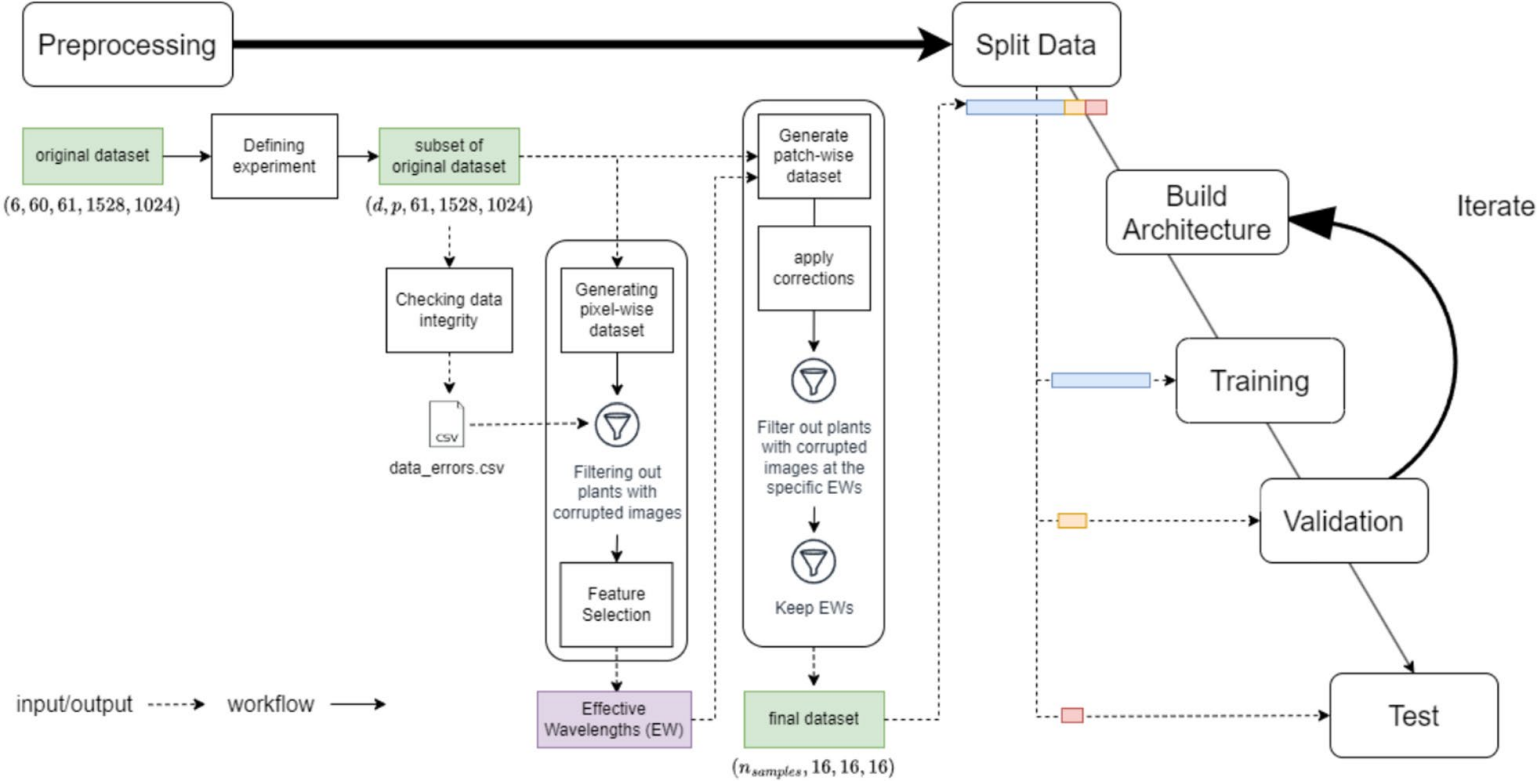
Experimental procedure workflow

A complete framework for validating data integrity and making the most of the available data was developed. This framework is visualized in the flowchart of Fig. 3. The original dataset is of dimensions $\left( {6, \, 60, \, 61, \, 1528, \, 1024} \right)$, since it includes 60 plant images, taken at 61 wavelengths and 6 time points (0, 2, 4, 6, 8, 10 DPI), each with dimensions of $1528 \times 1024$. The integrity of the data was checked (both computationally and manually), and the corrupted images were recorded in a CSV file of a predefined structure. Corrections were applied where possible, based on the assumptions that (i) a corrupted image at wavelength $\lambda$ can be replaced by a non-corrupted neighbouring image at $\lambda \pm 5$ nm and that (ii) a corrupted image at wavelength $\lambda$ can be replaced by the average of the two non-corrupted neighbouring images (i.e., the images at wavelengths $\lambda +10$ nm and $\lambda -10$ nm). These assumptions were not arbitrary but were based on the correlation of the images at neighbouring wavelengths, especially for wavelength range from 700 to $1000$ nm. A subset of the original dataset was defined for the experiment. *k* splits of the dataset were generated, and the Effective Wavelengths (EWs) were selected for each split, using only the plants with no corrupted images at any wavelength. The patch-wise dataset was then generated, using only the plants with no corrupted images at any of the 16 EWs. The patch-wise dataset comprises patches of dimensions $16 \times 16 \times 16$, taken from the area of the three upper leaves of a plant manually. This was the final set that was used as input in the classification. Data were then split into training, testing, and validation datasets, and the best architecture was selected using the trial-and-error approach. The algorithmic implementation of the aforementioned framework was developed in Python 3, using open-source Python libraries, and the dataset can be found at https://github.com/chadoulis/hyperspectralCube.^1^ In the following subsections, some aspects of the experimental framework are described in depth.

### Masking

**Fig. 4:**
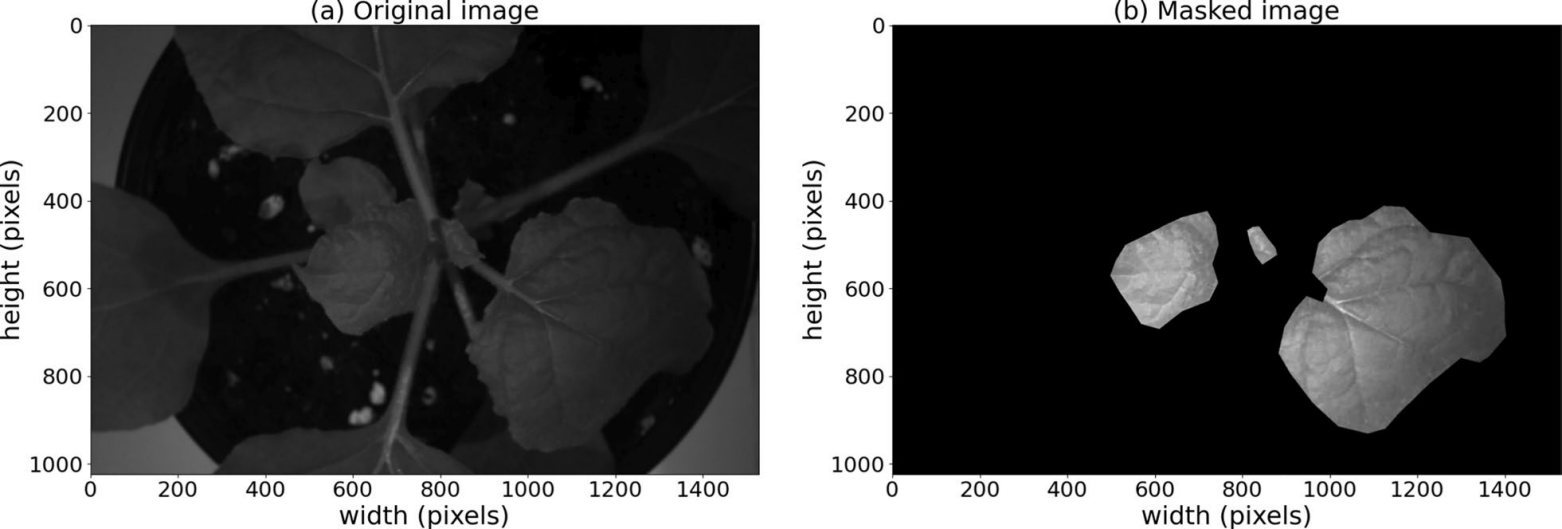
**a** The reflectance of plant 1 at 600 nm measured 2 DPI. **b** The reflectance of plant 1 at 600 nm measured 2 DPI and its mask overlay, which retains only pixels that solely represent leaf surface and belong to one of the 3 upper leaves of the plant

Raster masks were manually created for each plant hyperspectral cube using the QGIS software [39], in order to retain only pixels that solely represent leaf surface and belong to one of the 3 upper leaves of the plant, where plant viruses have systemically migrated via the phloem to induce symptoms. In Fig. 4, one can see a plant image and its masked version.

### Feature selection

We employ ERTs [25] to reduce hyperspectral dimensionality. The ERT is an ensemble method that grows multiple fully randomized decision trees, making it effective and computationally efficient for high-dimensional data. Below, we outline Tree Construction, Ensemble Generation, Feature Importance, and Selection Procedure.

#### Tree construction

Each individual tree in ERT is built by recursively partitioning the training set:

1. Random Feature Subset: At each node, a small subset of the available features is randomly chosen (or, in an extreme scenario, a single feature is selected).
2. Random Threshold: A cut-point is drawn uniformly at random from the range of values of the chosen feature. Rather than searching for the best threshold (as in standard decision trees), ERT inserts this randomized split without optimization.
3. Node Termination: Splitting continues until reaching a stopping criterion (e.g., a minimum node size) or until all samples in a node share the same class label.

#### Ensemble generation

The ERT algorithm aggregates $M$ such fully grown trees, each induced on the same training set. Due to the extreme randomness at each node, individual trees differ significantly from one another, decreasing overall variance when their outputs are combined (via majority vote or averaging).

#### Feature importance

Despite its random-split approach, ERT provides a measure of each feature’s relative importance. Specifically:

- For each tree $t_m$ in the ensemble, compute the total impurity reduction (e.g., Gini or variance reduction) contributed by each feature $f$. Here, $\Delta I_{m}(f)$ denotes the total decrease in impurity achieved by all splits on feature $f$ within tree $t_m$. For each node in tree $t_m$ where feature $f$ is used to make a split, calculate the impurity reduction resulting from that split and sum these reductions across the entire tree.
- Average these impurity reductions across all $M$ trees to obtain a global importance score:
  $$\begin{aligned} \text {Importance}(f) \;=\; \frac{1}{M} \sum _{m=1}^M \Delta I_{m}(f). \end{aligned}$$

Features are then ranked by descending importance.

#### Selection procedure

To reduce the initial set of hyperspectral bands:

1. Construct ERT: Build an ERT model on the full set of bands using the Tree Construction.
2. Compute Importance: Calculate the feature importance scores for each band.
3. Select Top Bands: Retain the most informative bands (e.g., top $K$ or those above a specified importance threshold).

### The patch-wise approach

Processing hyperspectral vegetation imagery is usually compromised by an imbalance between the limited availability of training samples and the high dimensionality of the imagery data, which is referred to as the Hughes phenomenon [23]. Different approaches have been proposed to solve this issue. The dominant approach is to extract only spectral information for each image pixel. This pixel-wise method assumes each pixel is represented by a one-dimensional vector of reflectance spectral measurements, and it is correspondingly labeled with the target. However, this approach neglects the spatial cues in leaf structures, such as veins or early lesion formations, that may be critical for detecting the onset of disease. To harness both the spectral and spatial attributes simultaneously, we propose a patch-wise method. The patch-wise approach allows us to capture a more comprehensive representation of the leaf surface by considering local spatial neighborhoods in addition to their spectral information. By doing so, we can leverage the inherent spatial patterns and structures that are indicative of early disease symptoms, which a pixel-wise method might overlook. The following paragraphs detail the methodology for extracting and classifying these patches effectively.

Let $\textbf{H} \in {\mathbb {R}}^{W \times H \times N_{\text {bands}}}$ denote a single hyperspectral image (also referred to as a *hypercube*), where $W$ is the image width (number of pixels along the horizontal axis), $H$ is the image height (number of pixels along the vertical axis), and $N_{\text {bands}}$ is the number of spectral bands. Each pixel location $(x, y)$, where $x \in \{1, 2, \dots , W\}$ and $y \in \{1, 2, \dots , H\}$, has an associated reflectance vector $\textbf{r}_{x,y} \in {\mathbb {R}}^{N_{\text {bands}}}$, such that:

$$\begin{aligned} \textbf{H}(x, y, :) = \textbf{r}_{x,y}. \end{aligned}$$

A binary mask $\textbf{M} \in \{0, 1\}^{W \times H}$ defines the *region of interest* (ROI) as:

$$\begin{aligned} \Omega = \left\{ (x, y) \in \{1, \dots , W\} \times \{1, \dots , H\} \ \bigg | \ \textbf{M}(x, y) = 1 \right\} . \end{aligned}$$

In this study, $\Omega$ corresponds to the leaf surfaces of the three upper leaves of the plant (see “Masking” section). Only pixels $(x, y) \in \Omega$ are eligible for patch extraction. To systematically extract nonoverlapping patches from the ROI, each patch $\textbf{P}_{i,j} \in {\mathbb {R}}^{p_x \times p_y \times N_{\text {bands}}}$ is defined with spatial dimensions $p_x \times p_y = 16 \times 16$ pixels. The extraction employs a stride $s = 16$ pixels in both the horizontal and vertical directions, ensuring that patches do not overlap and are adjacent to one another. Each patch can thus be defined and programmatically retrieved based on its bottom-left corner coordinates in the spatial dimensions, $\left( {i_k, j_l}\right)$, and its fixed size of $\left( 16,16,61\right)$. The coordinates defining each patch are defined along the horizontal and vertical axes as:

$$\begin{aligned} i_k= & 1 + k \cdot s, \quad k \in {\mathbb {N}}_0,\\ j_l= & 1 + l \cdot s, \quad l \in {\mathbb {N}}_0, \end{aligned}$$

where ${\mathbb {N}}_0 = \{0, 1, 2, \dots \}$. The indices $i_k$ and $j_l$ must satisfy the following constraints to ensure that each patch lies entirely within the ROI:

$$\begin{aligned} & i_k + p_x - 1 \le W, \quad \forall k \in {\mathbb {N}}_0,\\ & j_l + p_y - 1 \le H, \quad \forall l \in {\mathbb {N}}_0. \end{aligned}$$

**Fig. 5:**
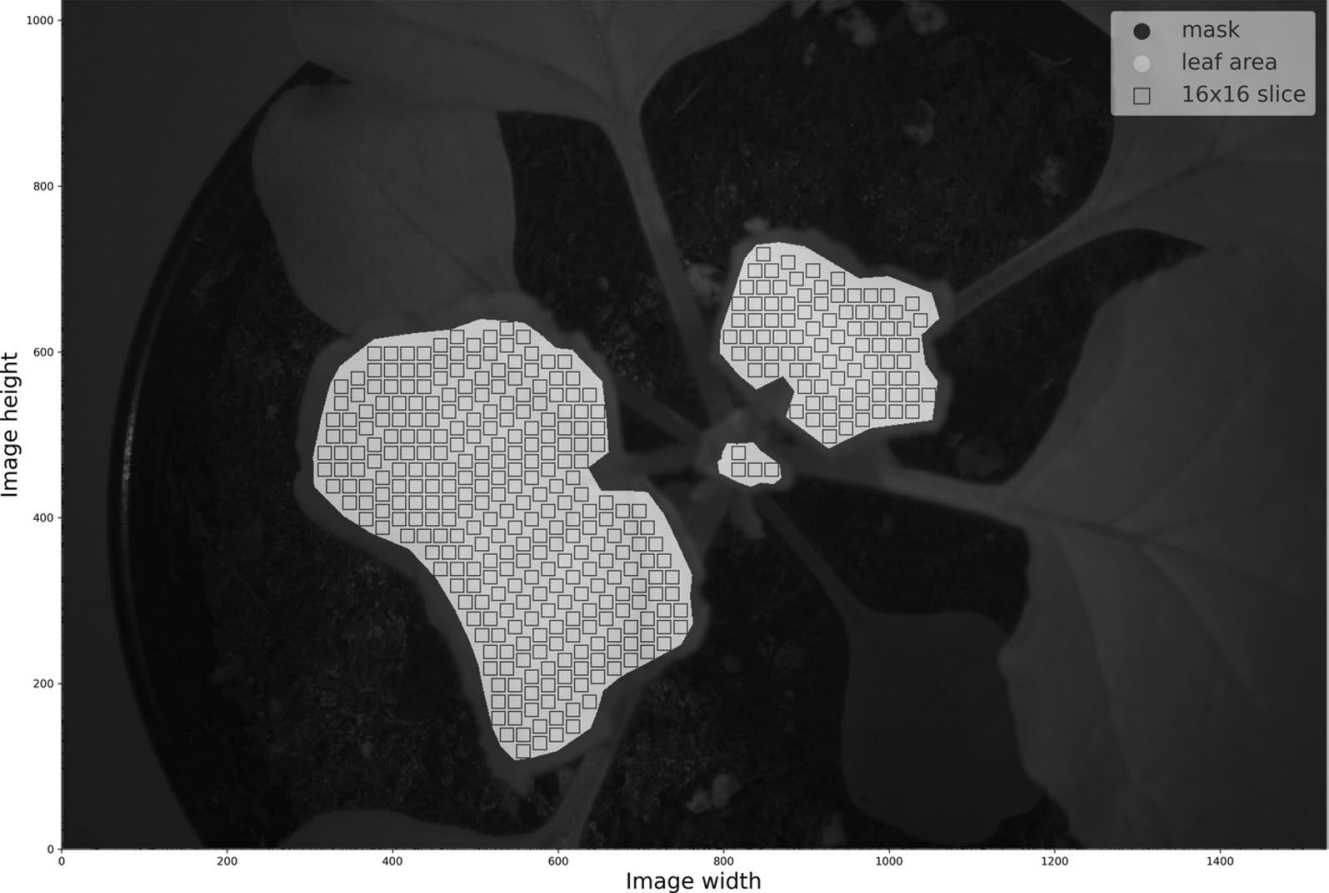
393 nonoverlapping square slices extracted out of the hyperspectral images of the plant with Plant ID = 2, measured on 11/09/2020

Each patch $\textbf{P}_{i,j}$ is formally defined as:

$$\begin{aligned} \textbf{P}_{i,j} = \textbf{H}\left[ i : i + p_x - 1,\; j : j + p_y - 1,\; 1 : N_{\text {bands}} \right] , \end{aligned}$$

where $i = i_k$ for some $k \in {\mathbb {N}}_0$, $j = j_l$ for some $l \in {\mathbb {N}}_0$, and the notation $\textbf{H}[a : b, c : d, u : v]$ extracts the sub-hypercube spanning pixels $a$ to $b$ horizontally, $c$ to $d$ vertically, and spectral bands $u$ to $v$. Given the stride $s = 10$ and patch size $16 \times 16$, patches are nonoverlapping and collectively cover the entire ROI $\Omega$ as shown in Fig. 5. Each patch captures a unique spatial-spectral sub-volume of the leaf surface, preserving both local spatial structures and comprehensive spectral information essential for accurate classification. The objective is to train a classifier function:

$$\begin{aligned} g : {\mathbb {R}}^{16 \times 16 \times N_{\text {bands}}} \rightarrow {\mathcal {C}}, \end{aligned}$$

where ${\mathcal {C}}$ represents the set of possible classes. In this study, the class set is defined as ${\mathcal {C}} = \left\{ \text {healthy}, \text {diseased} \right\}$. The classifier assigns a label $\hat{c}_{i,j} \in {\mathcal {C}}$ to each patch $\textbf{P}_{i,j}$ as follows:

$$\begin{aligned} \hat{c}_{i,j} = g\left( \textbf{P}_{i,j} \right) . \end{aligned}$$

This patch-wise labeling facilitates the generation of a detailed classification map over the leaf area, where each patch’s classification contributes to the spatial distribution of classes within the ROI.

This approach came as a result of observing that early viral or pathogen-induced symptoms often appear in small, localized regions-such as along veins or at lesion initiation points-rather than spreading uniformly across the leaf. A purely pixel-wise method would fail to capture these subtle local interactions, while large-scale spatial averaging might obscure or dilute them. By switching to patches, we effectively focus the classifier’s attention on regions that plausibly manifest early infection signs, while also addressing the high dimensionality and limited sample size characteristic of hyperspectral data. Specifically, subdividing each hyperspectral cube into smaller, nonoverlapping patches harnesses local spatial features (e.g., minor necrotic spots) alongside their spectral signatures, increasing the richness of input data beyond a simple pixel-wise approach. This strategy not only magnifies our training set size-vital when labeled samples are scarce-but also preserves critical neighborhood information in both spatial and spectral domains. Ultimately, classifying these smaller patches independently offers two major benefits: it facilitates earlier detection of subtle infection indicators, and it enables the generation of spatial heatmaps pinpointing where symptoms first emerge on the leaf surface. The result is a more targeted, robust, and interpretable framework for early and accurate disease detection.

### *k*-fold validation

Cross-validation is employed to assess the machine learning models. But instead of shuffling the samples randomly and splitting them into *k* groups, we performed the *k*-fold splitting at a plant level. This choice was made so that the test set comprised hyperspectral cubes from plants hitherto unseen by the model. The value of *k* is set equal to 3.

### 3D-CNNs

In this paper, we used a 3D-CNN architecture, a generalization of CNNs that operates in three dimensions. Several fundamental building blocks are used in a 3D-CNN to effectively extract features and make predictions. The Convolution3D layer is a key component of 3D-CNNs. It applies 3D convolution operations to the input volume to detect spatial patterns and features. MaxPooling3D layers are used for downsampling and to reduce the spatial dimensions of the feature maps produced by the Convolution3D layers. MaxPooling3D applies a 3D pooling operation to each feature map, reducing the size of the feature maps while retaining the most important information by taking the maximum value in each pooling region. Pooling helps to reduce the computational load and can provide some degree of translational invariance. Batch normalization layers in 3D-CNNs are crucial for the stabilization and acceleration of the training process. They work by normalizing the output of each convolutional layer, ensuring that the distribution of the activations remains consistent throughout the network. This normalization helps to reduce the internal covariate shift, leading to improved performance and faster convergence in 3D CNNs. Dropout layers in 3D-CNNs serve as a regularizing technique to prevent overfitting during training. By randomly setting a fraction of the input units to zero at each update during training, dropout layers introduce randomness that helps the network learn more robust features. This approach is particularly beneficial in 3D CNNs, which often deal with complex and high-dimensional data, ensuring the model generalizes better to unseen data.

### Loss function

The loss function used in model training was the categorical cross-entropy, a loss function that is used in multi-class classification tasks. Formally, it is designed to quantify the difference between two probability distributions and is given by:

1 $$\begin{aligned} \text {Loss} = -\sum \limits _{i=1}^{\text {output size}} y_{i} \log \hat{y}_{i} \end{aligned}$$

where $\hat{y}_i$ is the *i*-th scalar value in the model output, $y_i$ is the corresponding target value, and output size is the number of scalar values in the model output. During the training, the gradient of the loss function is computed with respect to each weight in the network. This gradient information is used to update the weights, aiming to reduce the loss in subsequent iterations.

### Classification accuracy evaluation metrics

The most obvious evaluation metric one could use to measure the performance of a model would be by calculating the proportion of correct predictions among the total number of samples tested, known as accuracy, and defined as:

2 $$\begin{aligned} \text {Accuracy} = \frac{TP+TN}{TP+FP+FN+TN} \end{aligned}$$

where *TP* is the number of True Positives, *TN* is the number of True Negatives, *FP* is the number of False Positives, and *FN* is the number of False Negatives. However, quantifying the success of a classification model is assessed with additional figures of merit. For the purposes of this paper, the *F*1-score is used, which was first introduced to evaluation tasks in information extraction at the Fourth Message Understanding Conference (MUC-4) in 1992 [40]. The *F*1 score is the harmonic mean of Precision and Recall, admitting a value in $\left[ {0,1} \right]$, i.e.,

3 $$\begin{aligned} F1 = 2 \cdot \frac{\text {Precision} \cdot \text {Recall}}{\text {Precision} + \text {Recall}} \end{aligned}$$

where Precision is the proportion of predicted positives that are truly positive,

4 $$\begin{aligned} \text {Precision} = \frac{TP}{TP+FP} \end{aligned}$$

and Recall is the proportion of actual positives that are correctly classified.

5 $$\begin{aligned} \text {Recall} = \frac{TP}{TP+FN} \end{aligned}$$

## Results

### Northern blot hybridization analysis

**Fig. 6:**
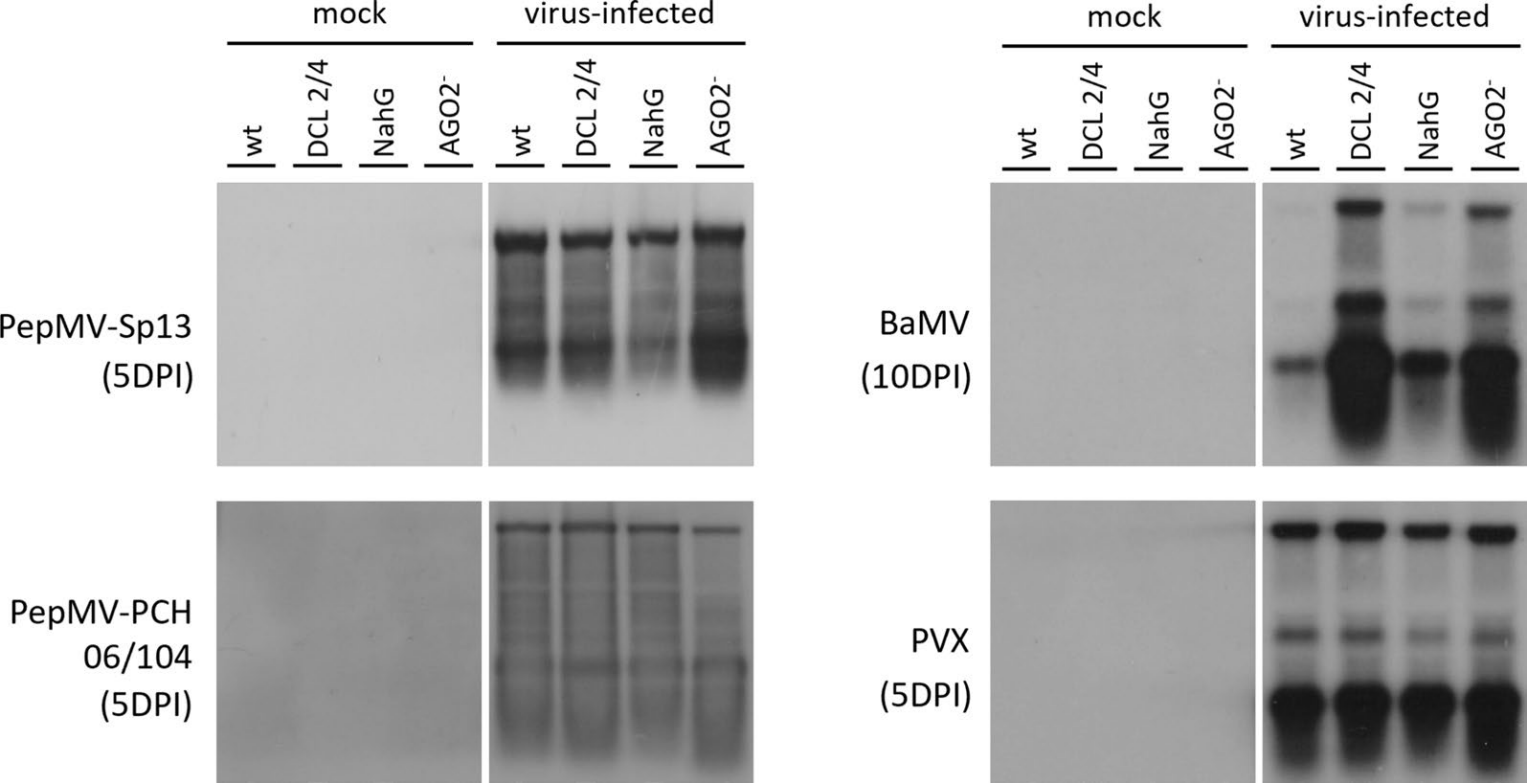
Northern blot hybridization analysis of total RNA extracts collected from four *N. benthamiana* (wt, DCL2/4, *NahG,* and *AGO2*) genotypes. Plants were mechanically-inoculated with 0.1*M* Tris-borate buffer (mock) or purified virions of one of the following potexviruses: *Pepino mosaic virus* (PepMV) SP13 (mild isolate), PepMV PCH 06/104 (severe isolate), *Bamboo mosaic virus* (BaMV) and *Potato virus X* (PVX). Total RNA extracts (1 to 5 μgr) from systemic leaves of mock- or virus-inoculated plants were collected and analyzed on northern blots hybridized with the respective DIG-labeled negative (–) strand coat protein probes

Potexvirus infection in all *N. benthamiana* genotypes at 5 and 10 DPI was verified by northern blot analyses using virus-specific DIG-labeled (–) RNA probesrepresenting the CP gene of each inoculated virus. As can be seen in Fig. 6, all probes specifically detected the genomic and sub-genomic viral RNAs in the infected plants, while no virus was detected in the total RNA extracts from mock-inoculated plants. Moreover, the PepMV mild and severe strain, and PVX viral RNA levels detected were higher at 5 DPI than 10 DPI, while the BaMV only accumulated to detectable levels at 10 DPI.

### Validating data integrity

**Fig. 7:**
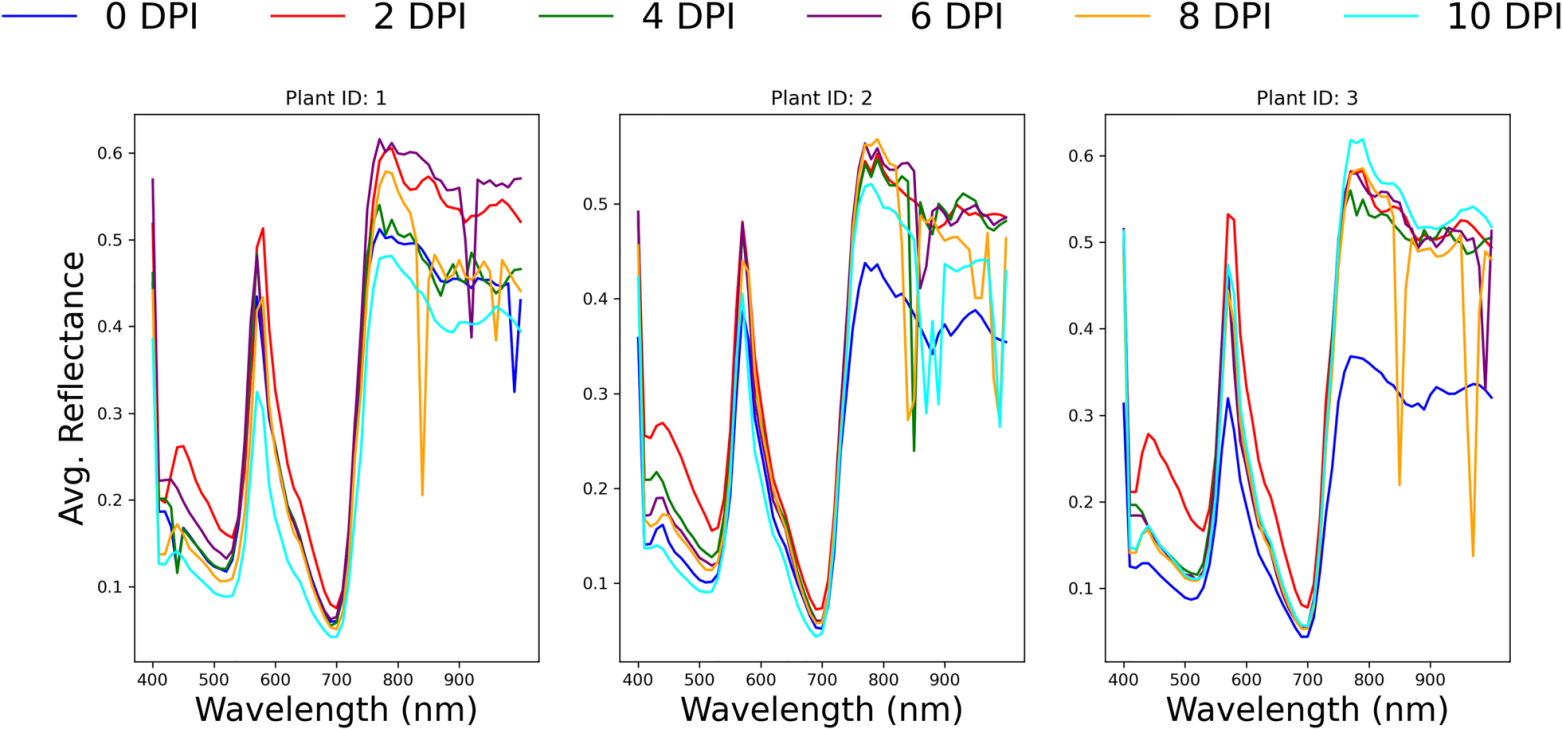
Spectral signature of three mock-inoculated plants, at 0, 2, 4, 6, 8, and 10 DPI

A series of testing functions were developed to ensure data integrity, i.e., to check the dimensions of the hyperspectral images and the masks and to check for missing or inconsistent data. As a result of this process, the hypercube of the plant 7, comprising images taken 8 DPI (19/09/2020), was completely removed from the dataset. The spectral signatures of all plants were examined across all dates, and a qualitative analysis was conducted as another step to ensure the integrity of the data. Figure 7 shows the average reflectance of the masked leaf surface of each of the three mock-inoculated plants of genotype WT across all wavelengths and at 0, 2, 4, 6, 8, and 10 DPI.

**Fig. 8:**
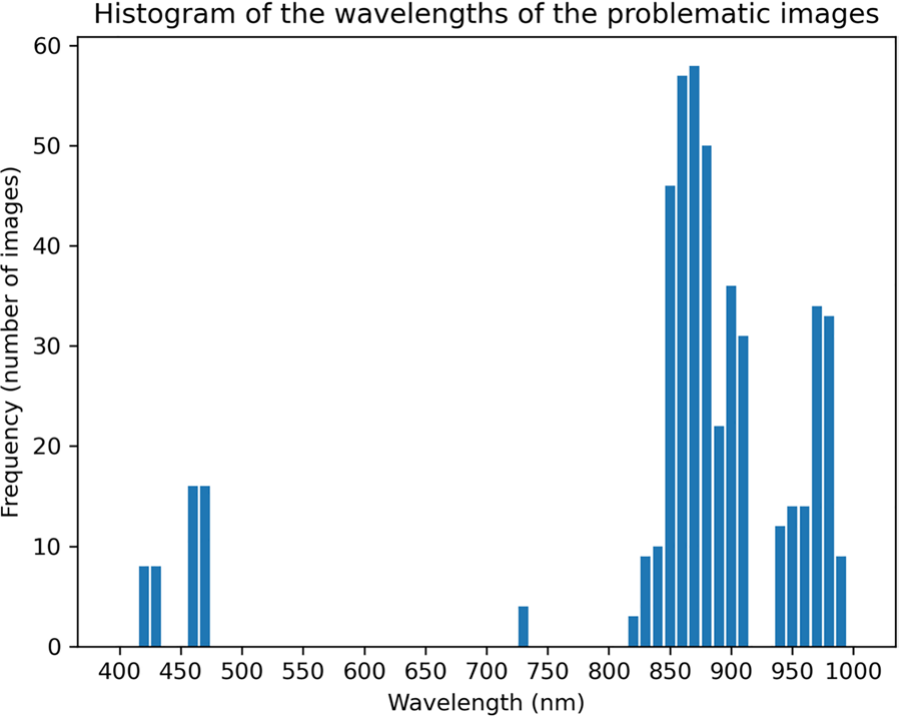
Histogram of wavelengths of the problematic images

**Fig. 9:**
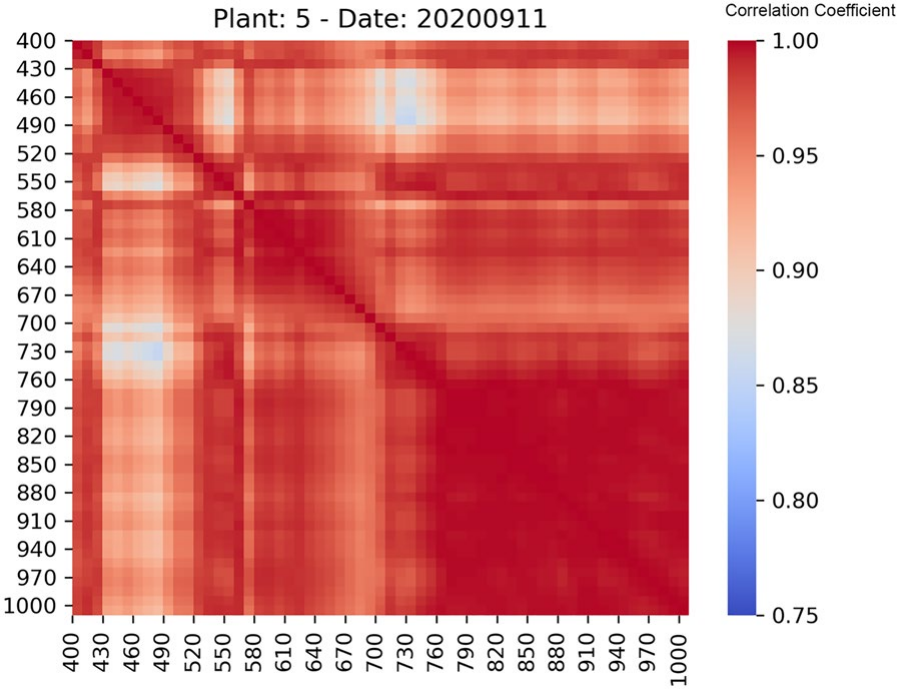
Correlogram of wavelengths

The spectral signature of a leaf surface from 400 to 1000 nm typically exhibits low reflectance in the blue and red visible regions due to strong absorption by chlorophyll with a distinctive “red edge” rising reflectance in the near-infrared region and variations influenced by leaf structure and health. The spectral signatures plotted appeared to comply with this general description. However, abrupt valleys appeared across all dates and at arbitrary wavelengths. After visually inspecting the images at the corresponding wavelengths, it became evident that large areas of these images were unreasonably darker than the rest of the image. This issue probably stemmed from instrumental limitations, calibration issues, or environmental factors that interfered with the measurements. Figure 8 shows the distribution of average reflectances across the wavelengths from 400 to 1000 nm of the problematic images in the original dataset. The abrupt valleys appearing at specific wavelengths are indicative of the issue. In addition, experimental evidence (see Fig. 9) indicated that neighboring bands, especially those in the area from 760 nm to 1000 nm, were highly correlated. In this context, the following preprocessing rules were employed:

- If an image at wavelength $\lambda$ is corrupted, and there is an image at $\lambda \pm 5$ nm, then this image can be used instead of the first one.
- If an image at wavelength $\lambda$ is corrupted, but its neighbouring images (i.e., the images at wavelengths $\lambda +10$ nm and $\lambda -10$ nm) are not, then this image can be replaced by the average of its neighboring images.
- Plants with images at one or more wavelengths that can’t be corrected are discarded.

**Fig. 10:**
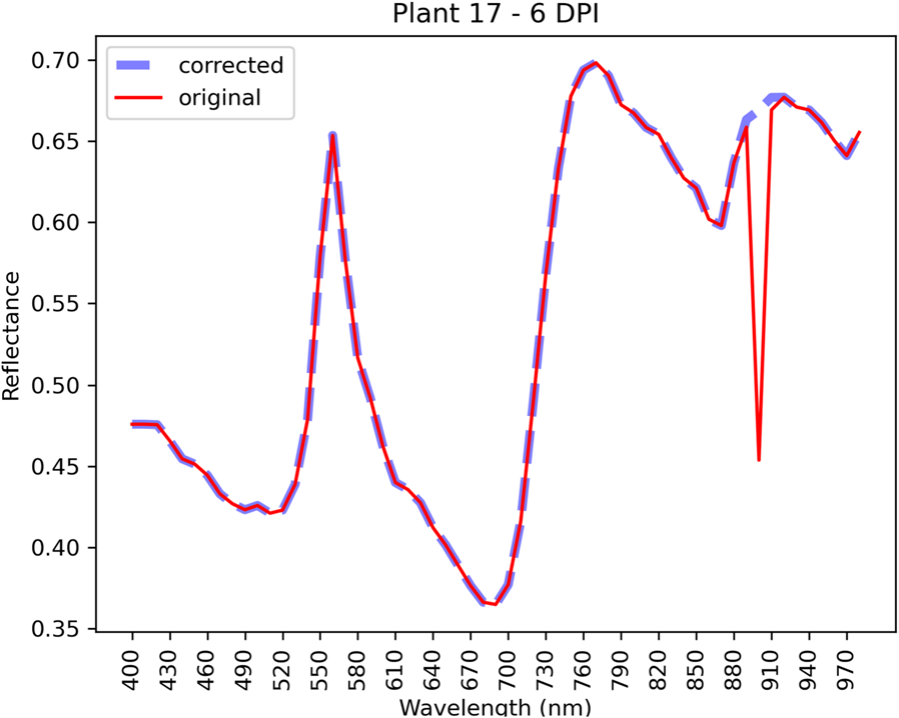
The average spectral signature of a $16 \times 16$ patch of Plant 17, 6 DPI, before and after applying the corrections

Figure 10 shows an example of the results when the corrections are applied. In this case, three images (at 890, 900, and 910 nm) were substituted with new ones.

### Pixel reflectance distribution across wavelengths

**Fig. 11:**
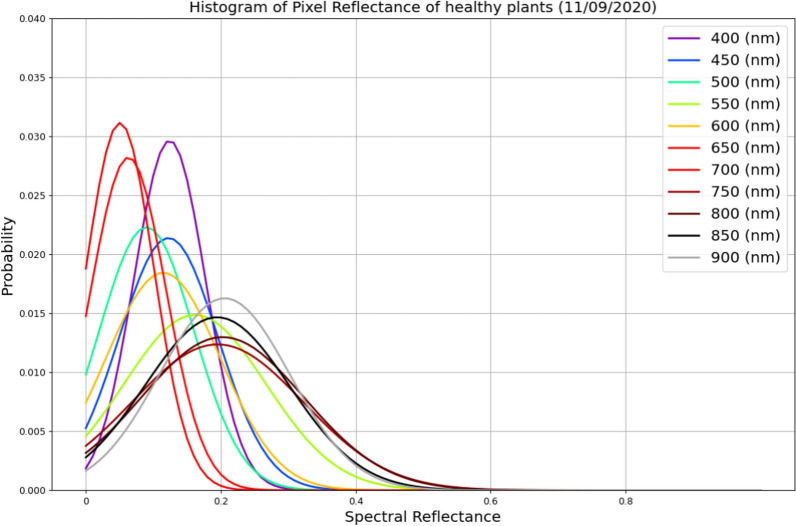
Histograms of reflectance of mock-inoculated plants for various wavelengths (11/09/2020)

The *t*-test has been proposed to differentiate mock-inoculated from infected plants. The *t*-test relies on a set of assumptions for correct interpretation and validity. For example, the dependent variable (i.e., the reflectance in each band) is assumed to be normally distributed. Examination of its distribution is shown in Fig. 11, where the histograms of reflectance of mock-inoculated plants at various wavelengths are plotted. Contrary to the claim in [17], this assumption did not seem to be valid for our dataset. In particular, the normality of the reflectance of each band was checked using the Shapiro-Wilk test, which showed that only the reflectance of the band corresponding to 790 nm was normally distributed.

### Classification between mock-inoculated and virus-infected plants

#### Train/test split and *k*-fold validation

**Fig. 12:**
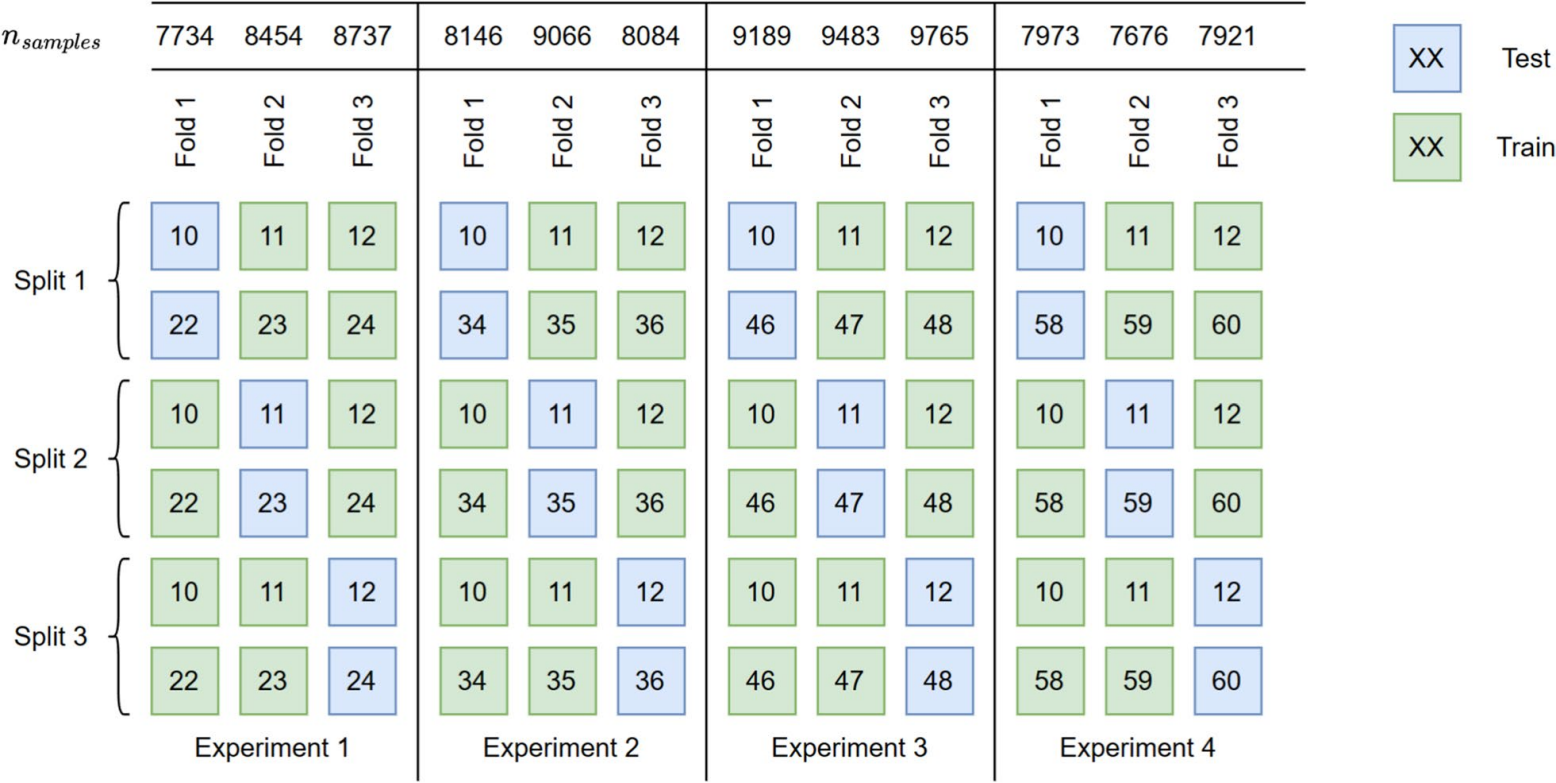
A summary of the threefold validation and train/test split scheme for the four experiments, and the number of samples ($n_{\text {samples}}$) in each fold

A comprehensive series of experiments was meticulously designed and executed to delve into the challenge of disease detection. The initial experiment focused on differentiating mock-inoculated plants from those infected with PepMV PCH 06/104, setting the stage for subsequent analyses. This was followed by the second experiment, which concentrated on distinguishing mock-inoculated plants from those infected with BaMV. The third experiment extended this investigation to plants infected with PVX. Finally, the fourth experiment aimed to discern mock-inoculated plants from those infected with PepMV SP13. In all four experiments, hyperspectral images of mock-inoculated plants and infected plants at 4, 6, and 8 DPI were used. To ensure a rigorous analysis, in each of these four experiments, the respective datasets were divided into 3 distinct folds. This approach guaranteed that each plant was uniquely assigned to a single fold, thereby maintaining the integrity of the data. The organization and results of these folds are summarized in Fig. 12.

#### EWs selection

EWs selection was performed, using the ERT algorithm at the pixel level and only for a subset of the test set of each fold, containing only the hyper-cubes with no corrupted images at any wavelength. This process yielded a set of EWs for each split of the dataset, as shown in Table 3. The redundant bands were discarded, and the new dimensions of each sample became equal to $16 \times 16 \times 16$.

**Table 3:** A summary of the EWs found for each of the 3 dataset splits of each of the four experiments

Experiment	Split	EWs (nm)
1	1	540, 550, 560, 680, 750, 820, 880, 890, 900, 910, 920, 930, 940, 950, 960, 970
	2	550, 560, 580, 740, 780, 800, 810, 870, 880, 890, 900, 910, 920, 940, 960, 970
	3	410, 440, 540, 550, 560, 580, 760, 770, 870, 880, 890, 900, 910, 920, 940, 950
2	1	410, 420, 430, 460, 480, 540, 550, 560, 570, 580, 590, 850, 880, 890, 900, 990
	2	410, 440, 460, 580, 670, 730, 740, 780, 810, 860, 890, 910, 920, 950, 960, 970
	3	410, 420, 430, 440, 450, 470, 490, 550, 560, 570, 750, 760, 770, 860, 880, 970
3	1	410, 430, 440, 450, 460, 470, 480, 490, 570, 740, 760, 880, 910, 940, 960, 980
	2	450, 550, 730, 750, 790, 800, 830, 860, 870, 900, 910, 920, 950, 970, 980, 990
	3	410, 430, 440, 450, 460, 470, 480, 490, 500, 510, 550, 820, 870, 900, 920, 990
4	1	760, 770, 780, 790, 800, 810, 820, 840, 850, 870, 880, 890, 900, 910, 930, 960
	2	430, 550, 740, 750, 810, 840, 880, 890, 900, 910, 920, 930, 970, 980, 990, 1000
	3	400, 420, 540, 550, 580, 740, 780, 810, 890, 900, 920, 930, 940, 950, 960, 970

#### Training

The 3D-CNN was compiled with the Adam optimizer and binary cross-entropy as the loss function, specifying accuracy as the primary performance metric. Up to 12 training epochs were conducted, with each epoch involving mini-batch iterations across the full training set. A batch size of 32 was employed to balance computational efficiency and stable gradient estimates. Throughout training, the model’s loss and accuracy were tracked, and overfitting was mitigated by incorporating dropout (rate = 0.5).

#### 3D-CNN architecture

**Fig. 13:**
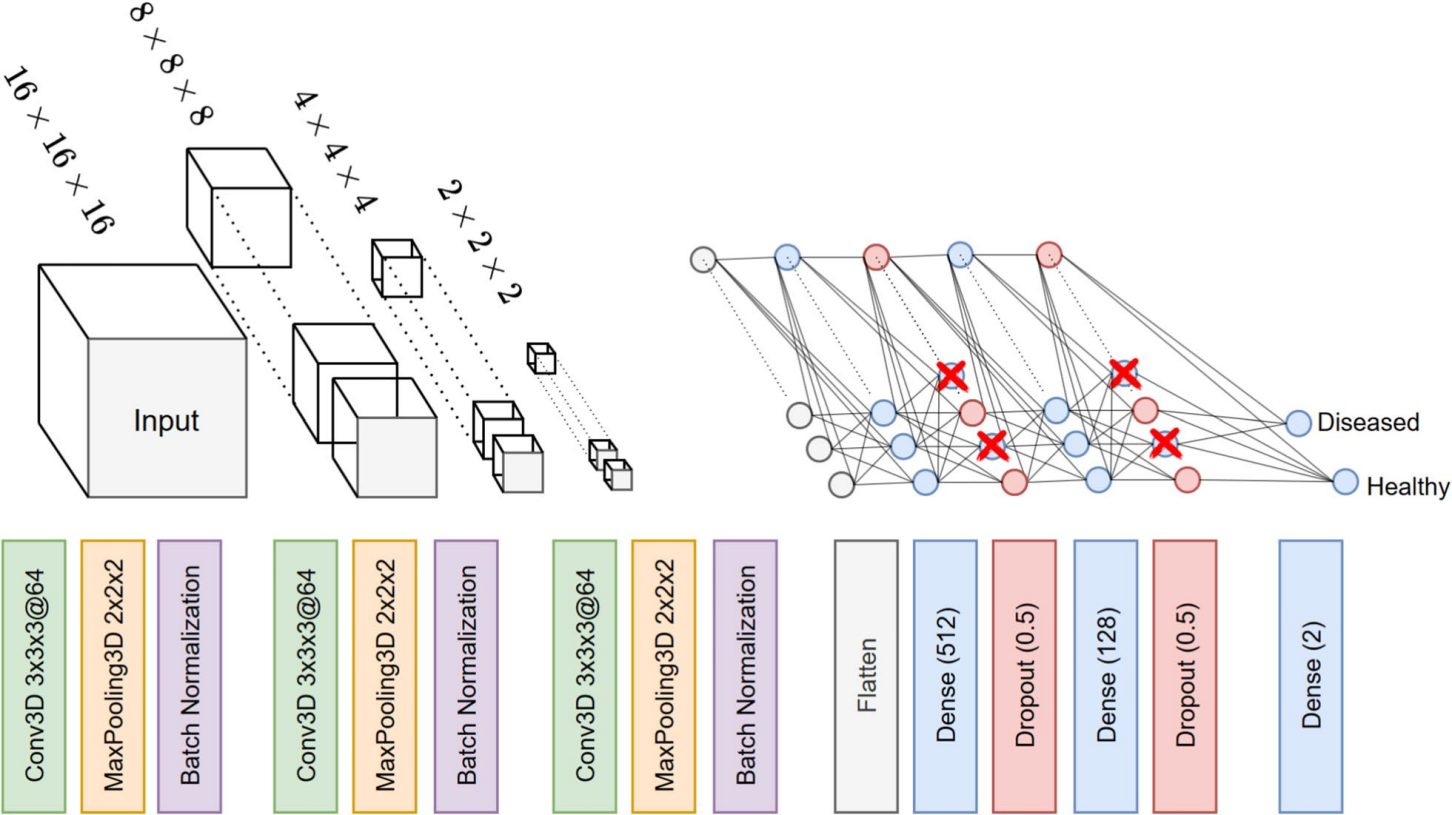
The 3D-CNN Architecture. For the Convolutional layers, the notation $3 \times 3 \times 3$@64 denotes a convolutional layer comprising 64 filters of dimension $3 \times 3 \times 3$, for the Dense layer, the number in the parenthesis denotes the number of nodes and for the Dropout layers, the number in the parenthesis denotes the probability a neuron of the previous layer being dropped

In the proposed 3D-CNN architecture, the initial layer, equipped with 64 filters, extracts spectral-spatial features from the input, which are then sub-sampled by a subsequent MaxPooling3D layer to reduce dimensionality while preserving essential information. To enhance the network’s training stability and efficiency, batch normalization layers follow each convolutional and pooling step, normalizing the inputs to each layer. The architecture further includes additional Conv3D layers, each followed by MaxPooling3D and batch normalization, to deepen the feature extraction process. Then, the network transitions to a fully connected regime, beginning with a Flatten layer to reshape the 3D feature maps into a 1-D vector. This vector is then passed through dense layers, incorporating dropout for regularization to mitigate overfitting. The network culminates in a final dense layer with two output units, indicating its utility for binary classification tasks. Overall, this 3D-CNN architecture has 552, 450 parameters, of which 552, 066 are trainable (Fig. 13).

#### Classification accuracy

**Fig. 14:**
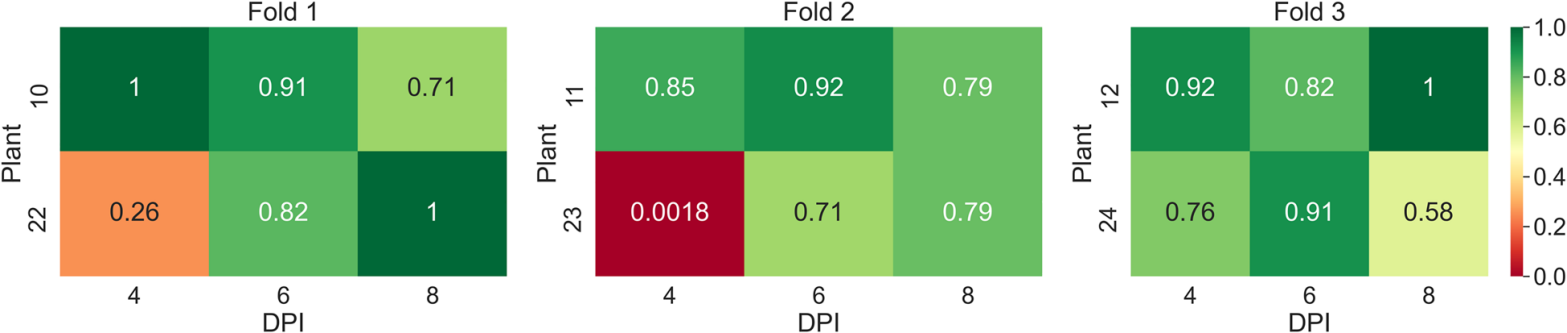
Heatmap of accuracy per plant, 4, 6, and 8 DPI, across the threefolds of the first experiment (mock-inoculated-PCH 06/104)

In the first experiment, the task of classifying samples into mock and PepMV SP13-inoculated plants was investigated using *AGO2* mutants. The average overall accuracy was found to be equal to 0.82. The confusion matrix presented in Table 4 provides a comprehensive overview of the classification model’s performance across the 3 splits. For Split 1, precision, recall, and $F_1$ scores for the mock-inoculated class were relatively high, achieving an overall accuracy of 0.84. Split 2 showed a decrease in performance with the lowest overall accuracy of 0.74, marked by notably lower precision and $F_1$ scores for the PepMV SP13 predictions. Conversely, Split 3 exhibited the best performance with the highest overall accuracy of 0.87, and impressive precision and $F_1$ scores, especially for the mock-inoculated class. Across the splits, the model demonstrated stronger predictive capabilities for the mock-inoculated class compared to the PepMV SP13 class, culminating in an average overall accuracy of 0.82 for the entire experiment. In Fig. 14, one can see the classification accuracies per plant, 4, 6, and 8 DPI, across the threefolds of the first experiment.

**Table 4:** Confusion matrices for tests performed across the 3 splits of the dataset of the first experiment (mock-inoculated-PepMV SP13) along with the corresponding recall, precision, *F*1-scores, and overall accuracies

	Actual mock-inoculated	Actual PepMV SP13	Precision	$F_1$ score	Overall accuracy
Split 1					
Predicted mock-inoculated	2744	364	0.88	0.88	0.84
Predicted PepMV SP13	415	1286	0.76	0.78	
Recall	0.87	0.78	–	–	
Split 2					
Predicted mock-inoculated	2974	486	0.86	0.81	0.74
Predicted PepMV SP13	931	1143	0.56	0.70	
Recall	0.76	0.70	–	–	
Split 3					
Predicted mock-inoculated	3320	303	0.92	0.90	0.87
Predicted PepMV SP13	461	1584	0.77	0.84	
Recall	0.88	0.84	–	–	
Average					0.82

In the second experiment, the task of classifying samples into mock and PepMV PCH 06/104-inoculated plants was investigated using *AGO2* mutants. The average overall accuracy was found to be equal to 0.87. In Table 5, one can see the corresponding confusion matrices for each of the 3 splits.

**Table 5:** Confusion matrices for the tests performed across the 3 splits of the dataset of the second experiment (mock-inoculated-PCH 06/104) along with the corresponding recall, precision, $F_1$-scores, and overall accuracies

	Actual mock-inoculated	Actual PCH 06/104	Precision	$F_1$ score	Overall accuracy
Split 1					
Predicted mock-inoculated	1590	661	0.71	0.81	0.86
Predicted PCH 06/104	74	3034	0.98	0.82	
Recall	0.96	0.82	–	–	
Split 2					
Predicted mock-inoculated	1990	272	0.88	0.84	0.86
Predicted PCH 06/104	506	2954	0.85	0.92	
Recall	0.80	0.92	–	–	
Split 3					
Predicted mock-inoculated	1359	118	0.92	0.82	0.89
Predicted PCH 06/104	468	3155	0.87	0.96	
Recall	0.74	0.96	–	–	
Average					0.87

**Fig. 15:**
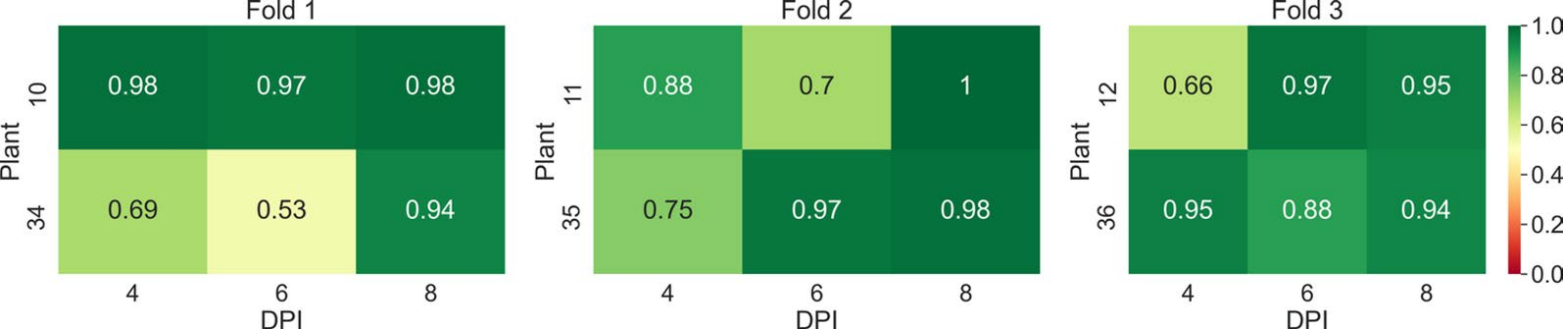
Heatmap of accuracy per plant, 4, 6, and 8 DPI, across the threefolds of the second experiment (mock-inoculated-PCH 06/104)

In Fig. 15, one can see the classification accuracies per plant, 4, 6, and 8 DPI across the threefolds of the second experiment.

In the third experiment, the task of classifying samples into mock and BaMV-inoculated plants was investigated using *AGO2* mutants. The average overall accuracy was found to be equal to 0.78. In Table 6 one can see the corresponding confusion matrices for each of the 3 splits.

**Table 6:** Confusion matrices for tests performed across the 3 splits of the dataset of the third experiment (mock-inoculated-BaMV) along with the corresponding recall, precision, $F_1$-scores, and overall accuracies

	Actual mock-inoculated	Actual BaMV	Precision	$F_1$ score	Overall accuracy
Split 1					
Predicted mock-inoculated	2975	589	0.83	0.80	0.78
Predicted BaMV	911	2197	0.71	0.79	
Recall	0.77	0.79	–	–	
Split 2					
Predicted mock-inoculated	2900	499	0.85	0.80	0.80
Predicted BaMV	921	2539	0.73	0.83	
Recall	0.76	0.84	–	–	
Split 3					
Predicted mock-inoculated	1837	1253	0.60	0.71	0.78
Predicted BaMV	244	3379	0.93	0.73	
Recall	0.88	0.73	–	–	
Average					0.78

**Fig. 16:**
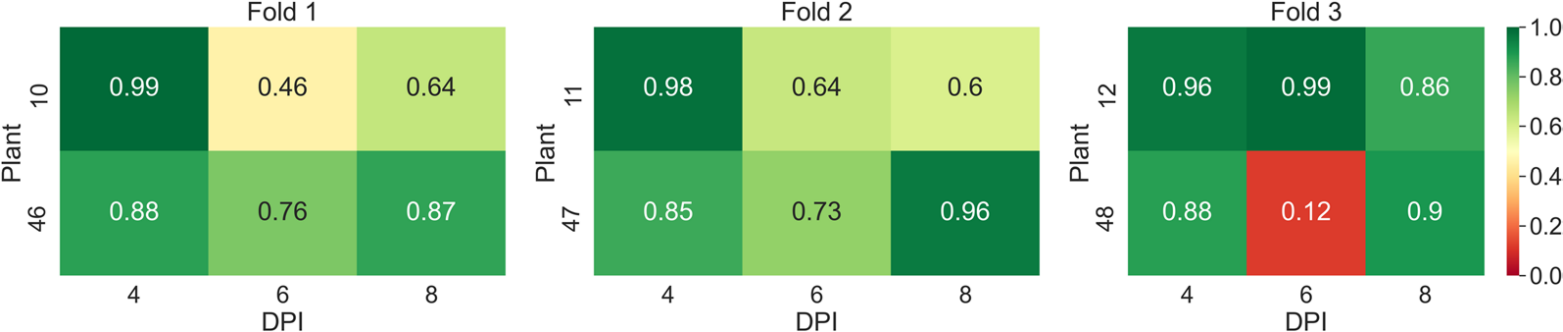
Heatmap of accuracy per plant, 4, 6, and 8 DPI, across the threefolds of the third experiment (mock-inoculated-BaMV)

In Fig. 16 one can see the classification accuracies per plant, 4, 6, and 8 DPI, across the threefolds of the third experiment.

In the fourth experiment, the task of classifying samples into mock and PVX-inoculated plants was investigated using *AGO2* mutants. The average overall accuracy was found to be equal to 0.86. In Table 7, one can see the corresponding confusion matrices for each of the 3 splits.

**Table 7:** Confusion matrices for tests performed across the 3 splits of the dataset of the third experiment (mock-inoculated-PVX) along with the corresponding recall, precision, $F_1$-scores, and overall accuracies

	Actual mock-inoculated	Actual PVX	Precision	$F_1$ Score	Overall accuracy
Split 1					
Predicted mock-inoculated	1785	648	0.73	0.80	0.84
Predicted PVX	261	2847	0.92	0.81	
Recall	0.87	0.81	–	–	
Split 2					
Predicted mock-inoculated	1826	169	0.92	0.83	0.87
Predicted PVX	562	2898	0.84	0.94	
Recall	0.76	0.94	–	–	
Split 3					
Predicted mock-inoculated	1183	365	0.76	0.78	0.87
Predicted PVX	300	3323	0.92	0.90	
Recall	0.80	0.90	–	–	
Average					0.86

**Fig. 17:**
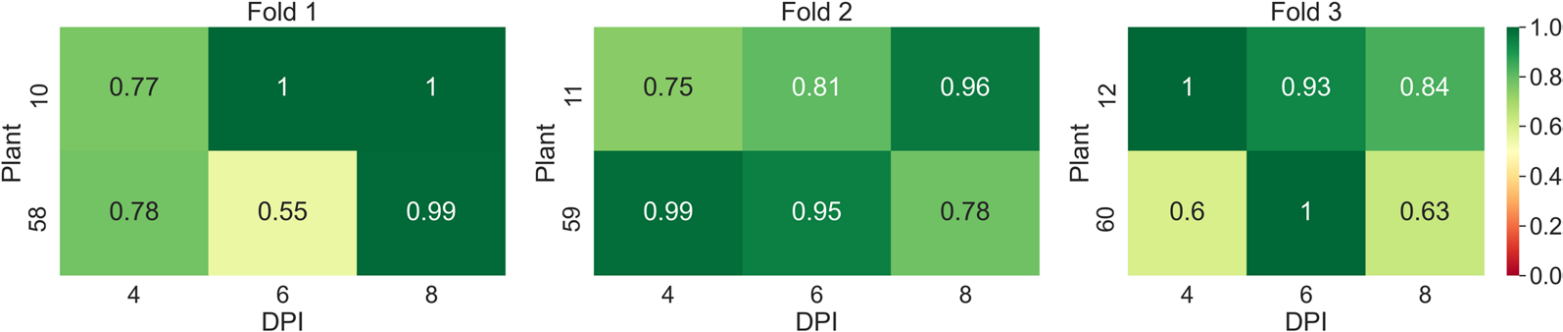
Heatmap of accuracy per plant, 4, 6, and 8 DPI, across the threefolds of the fourth experiment (mock-inoculated-PVX)

In Fig. 17, one can see the classification accuracies per plant, 4, 6, and 8 DPI, across the threefolds of the fourth experiment.

### Earliness of detection

**Fig. 18:**
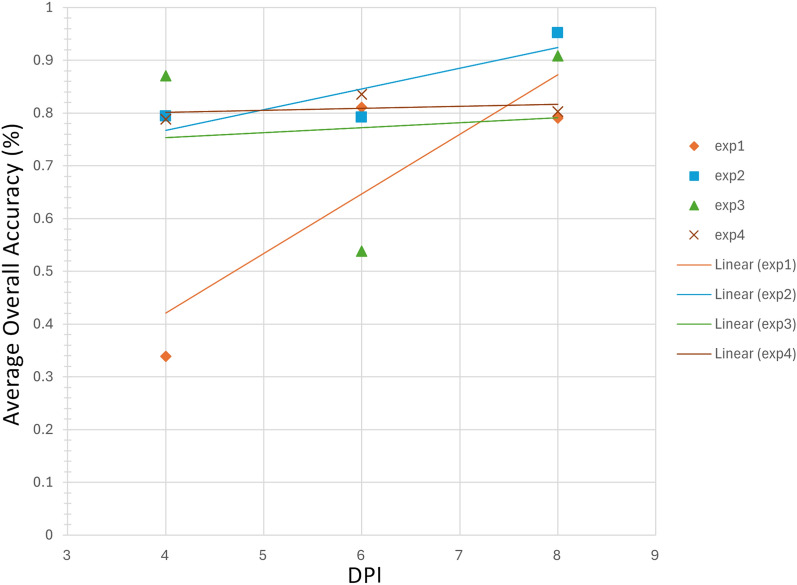
Correlation between average classification accuracy and DPIs, for all 4 experiments, during the time course of *Potexvirus* infection at 2, 4, 6, and 8 DPI

The classification results demonstrated a significant correlation between the overall classification accuracies and the progression of the disease over time. Figure 18 shows a scatter plot illustrating the trend of average classification accuracy over time (measured in DPI) for the four different experiments (exp1, exp2, exp3, and exp4), quantified by the coefficient of determination $R^{2}$ values for each experiment’s trendline. Exp1 and exp2, with $R^{2}$ values of 0.7159 and 0.7392, respectively, showed strong positive trends in classification accuracy as time progressed, indicating effective disease detection as the infection developed. These high $R^{2}$ values suggest that the models used in exp1 and exp2 reliably predicted disease states with increasing accuracy over time. Conversely, exp3 and exp4 exhibited significantly lower $R^{2}$ values of 0.0088 and 0.0934, indicating very weak trends in classification accuracy. For experiments 1, 2, and 4, classification accuracy reached 0.8 at 6 DPI, whereas for experiment 3, accurate classification was not possible until 8 DPI. This finding agrees with the northern blot analysis, which indicated a slower disease evolution for BaMV.

### Sensitivity analysis

**Fig. 19:**
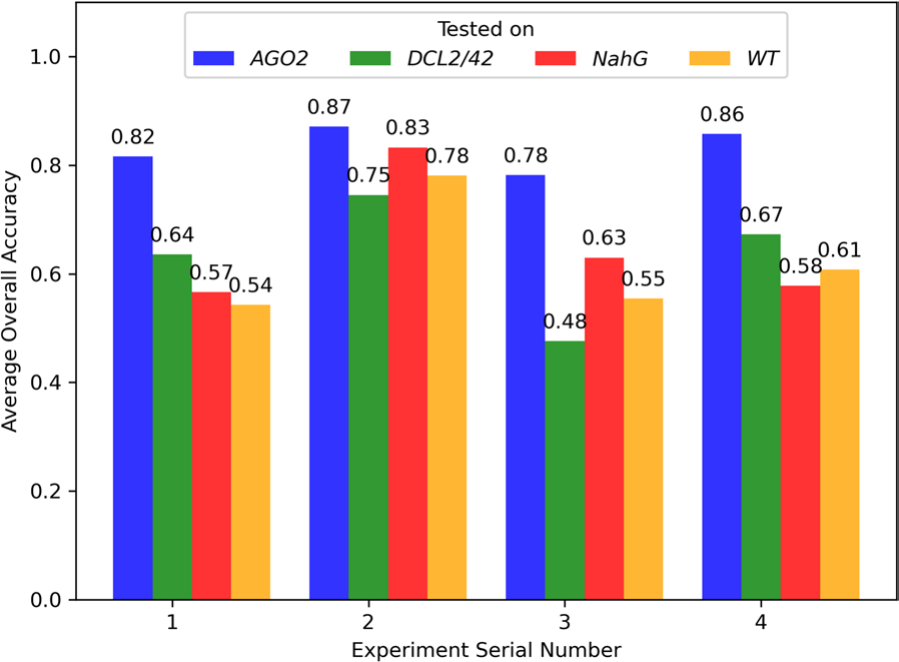
Average overall accuracies of the models trained on images from plants of genotype *AGO2* and tested on images from *AGO2*, *DCL2/4*, *NahG,* and WT, across the four experiments, i.e., mock-inoculated vs PepMV SP13, mock-inoculated vs PCH 06/104, mock-inoculated vs BaMV, and mock-inoculated vs PVX

In order to investigate how well the algorithm could generalize, the twelve models that were trained on plants of genotype *AGO2*, were tested on plants of genotype *DCL2/4*, *NahG,* and WT. Figure 19 shows a comparison of the average overall accuracies. As expected, the models that were trained on the plants of genotype *AGO2*, performed less well when tested on plants of a different genotype. In particular, the average classification accuracies of the models tested on plants of genotype *DCL2/4* ranged from 0.48 to 0.67, the average classification accuracies of the models on plants of genotype *NahG* ranged from 0.57 to 0.83, and the average classification accuracies of the models on plants of the wild type ranged from 0.54 to 0.78. It is evident that the model for classifying between mock-inoculated and PepMV PCH 06/104 generalized better than the others across all genotypes.

### Benchmarking against related work

Using our dataset as a benchmarking dataset, we compared the performance of the proposed ERT+3DCNN model with (i) the best-performing successive projection algorithm + extreme gradient boosting (SPA+XGBoost) approach [19] and (ii) a hybrid approach employing the ERT algorithm for feature selection and XGBoost for classification. The SPA is a forward selection technique that iteratively selects relevant spectral bands to minimize collinearity, thereby enhancing model interpretability. In both benchmarking approaches, we employed XGBoost, an optimized gradient boosting library, with hyperparameters optimized via GridSearchCV. The hyperparameter search included the number of estimators (100, 200, 300), maximum depth (3, 5, 7), learning rate (0.001, 0.05, 0.1), subsample ratio (0.7, 0.8, 0.9), gamma (0, 0.1, 0.2), and minimum child weight (1, 5, 10). The optimal parameters identified through GridSearchCV were then employed to benchmark against our proposed ERT+3DCNN model. This methodological alignment ensures a fair and comprehensive comparison, highlighting the efficacy of ERT+3DCNN in capturing complex spatial-spectral patterns and enhancing classification performance in hyperspectral-based plant viral disease detection.

**Fig. 20:**
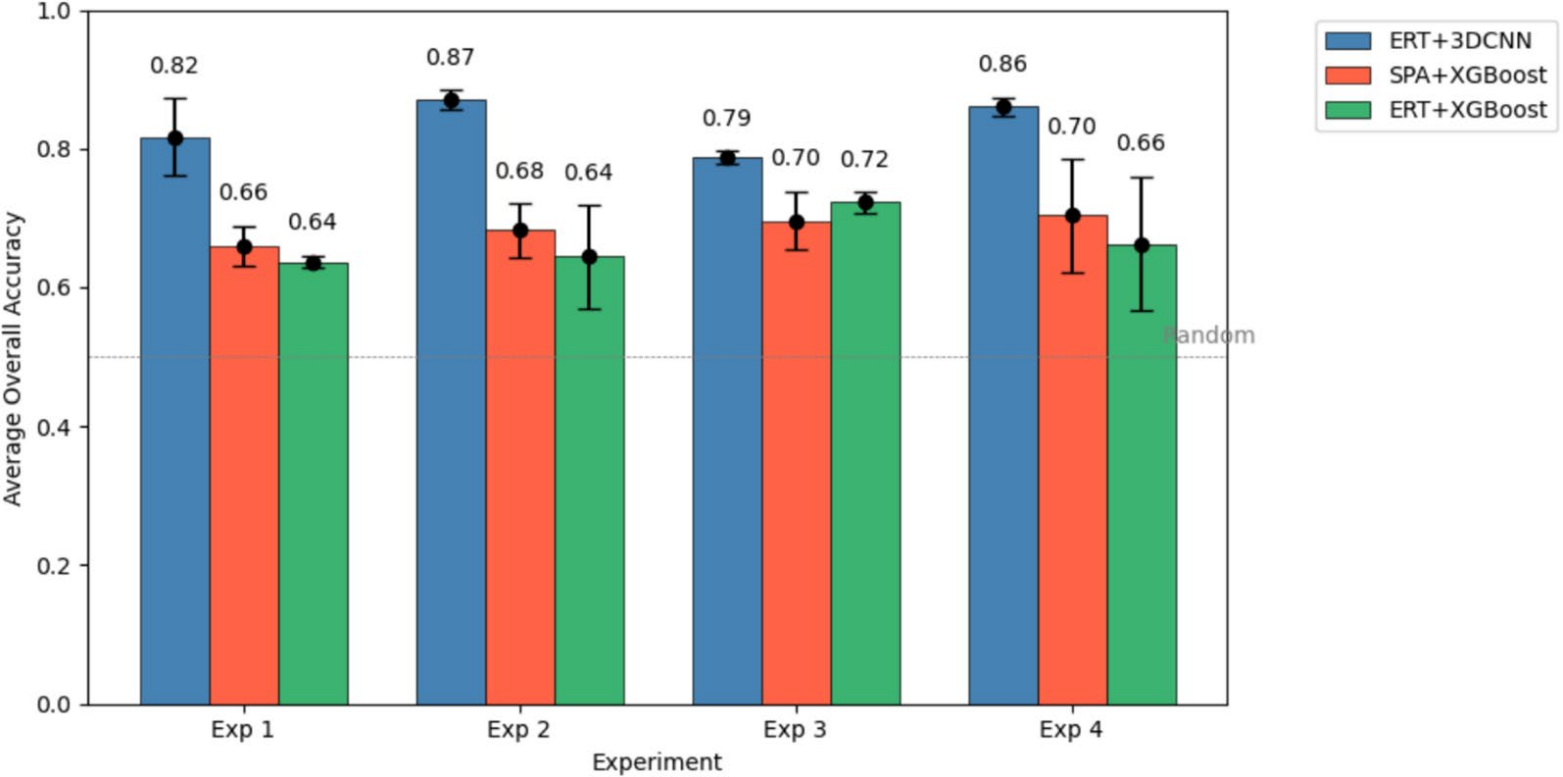
Average Overall accuracies (across the threefolds) of the models trained using the proposed ERT+3DCNN approach and the state-of-the-art SPA+XGBoost method across four experiments

Overall accuracies achieved by the SPA+XGBoost approach range from 0.66 to 0.7, and accuracies achieved by the hybrid ERT+XGBoost approach range from 0.64 to 0.72. The results, summarized in Fig. 20, demonstrate that the ERT+3DCNN, which achieves overall accuracies ranging from 0.79 to 0.87, consistently achieves higher classification accuracy compared to SPA+XGBoost across all experimental setups. This consistent improvement suggests that the ERT+3DCNN model is better suited for capturing complex patterns in the dataset, offering enhanced performance in distinguishing between the various classes. It is worth mentioning that for Experiment 2, the overall accuracy the proposed method achieves on plants of hitherto unseen genotypes (Fig. 19) is higher than the overall accuracy achieved by the SPA+XGBoost when tested on plants of the genotype it was trained with. Such findings highlight the potential of ERT+3DCNN as a competitive alternative to existing state-of-the-art algorithms in the domain.

## Discussion

The usefulness of 3D-CNNs has been demonstrated for early disease detection using hyperspectral imagery. It has also been shown that employing a patch-wise approach may be a practical and efficient way to generate a plethora of spectral-spatial training samples using only a few hyperspectral plant images. In the bulk of the literature, researchers tend to use either spectral or spatial representations to train models for leaf disease detection. On the contrary, the proposed method addresses the problem of leaf disease detection in a spectral-spatial fashion. The novelty of the proposed method lies in the fact that by taking nonoverlapping slices of a leaf’s hyperspectral image, one can train a robust classifier using only a few hyperspectral images, out of which plenty nonoverlapping square hyperspectral slices can be extracted.

**Fig. 21:**
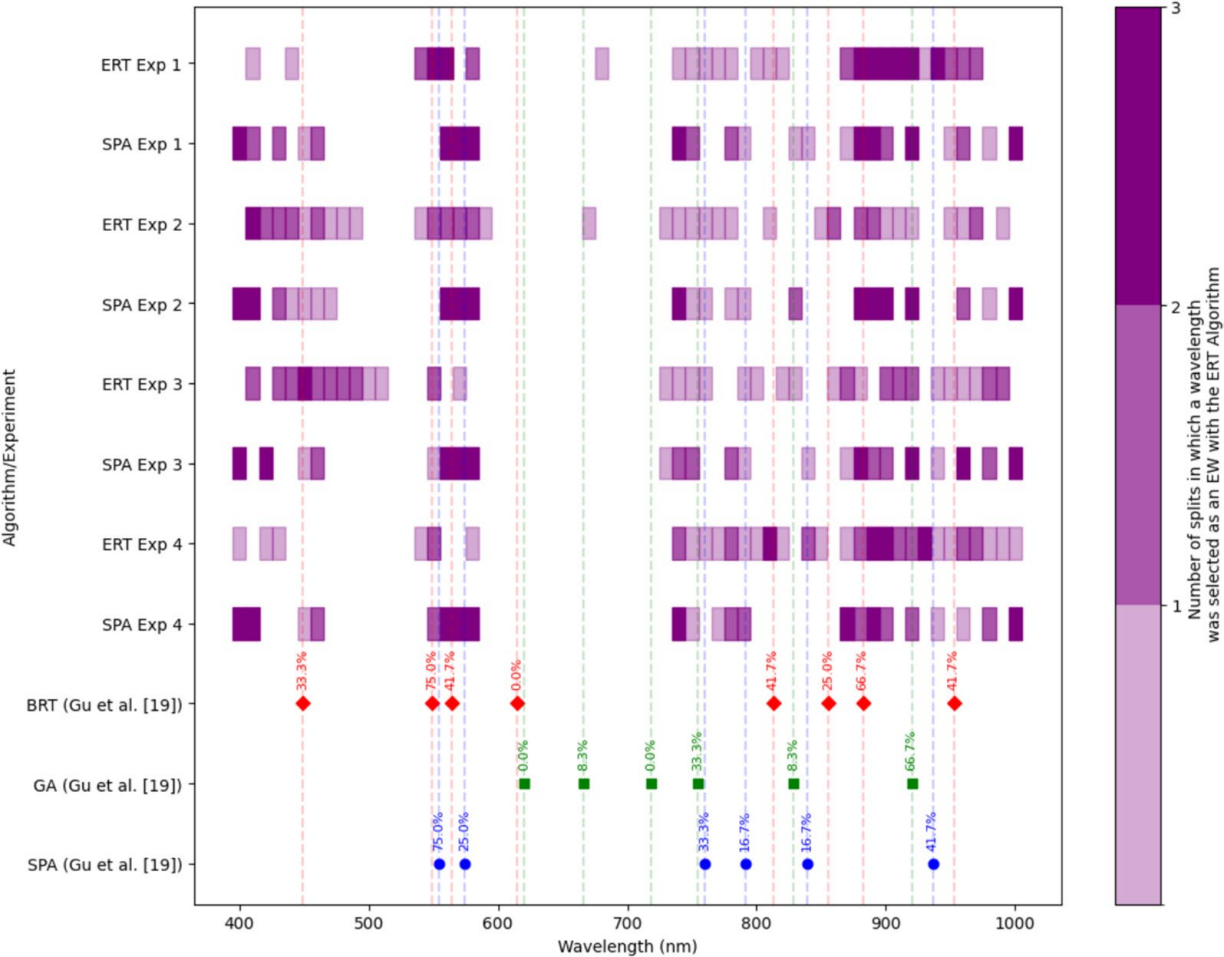
Comparison between (i) the EWs in our paper using the ERT algorithm, (ii) the EWs using the SPA algorithm proposed by Gu et al. on our dataset, and (iii) the EWs using SPA, GA, and BRT as in [19]. The vertical dashed lines represent the bands chosen by SPA, GA, and BRT in Gu’s work, with each point annotated by the percentage overlap with ERT-selected bands across the four experiments. The purple shading indicates the number of ERT dataset splits in which a band was selected, with darker shades representing higher frequencies of selection

The comparison between (i) the selected EWs in our paper using the ERT algorithm, (ii) the selected EWs using the SPA algorithm proposed in [19] on our dataset, and (iii) the selected EWs originally proposed in [19] shows some overlap in key spectral regions, particularly in the visible (around 550 nm), as shown in Fig. 21. This comes as no surprise since when a plant is under stress, such as during viral infections, changes occur in the plant’s physiological state, and these changes may result in altered chlorophyll content or degradation, which affects the reflectance in the visible range, particularly around 550 nm. Also, a large percentage of the selected EWs lay in the spectral region beyond 700 nm. This result is consistent with findings of previous studies. Zhang et al. demonstrated that the Near-Infrared (NIR) range is significantly more informative than the visible region for detecting late blight disease in tomato crops [41], while Gu et al. [19] observed that the NIR region (780–1000 nm) in the reflectance spectra, identified through feature selection methods, is more informative for distinguishing between *TSWV*-infected and mock-inoculated tobacco leaves than is the visible range.

The overall classification accuracies achieved could be juxtaposed to the state-of-the-art. In particular, in this study, the average overall accuracies achieved at detecting four different viral diseases caused by four *Potexvirus*es range from 0.78 to 0.87, while the accuracies reported by Gu et al. [19], detecting TSWV infection, ranged from 0.555 to 0.852. However, comparing classification accuracies achieved in different research efforts is not trivial and may be misleading. Since there are no publicly available and widely adopted plant disease detection benchmark datasets, it is not possible to faithfully compare the classification accuracies reported in the literature. This is the reason it was deemed necessary to reproduce the SPA+XGBoost proposed by Gu and benchmark it against the proposed approach, using our dataset as the benchmark dataset.

Throughout the experiments, the influence of a wide variety of parameters on the results became apparent. For example, in some cases, the square slices taken over the central vein of a leaf belonging to a mock-inoculated plant are systematically classified incorrectly as belonging to diseased plants. This highlights the fact that this method not only achieves good classification accuracies but also reinforces the explainability of the results.

The dataset size is also crucial for robust model development. We believe our dataset is sufficient to support the conclusions drawn in this study for several reasons. First, although the total number of plants (biological replicates) may seem modest, each hyperspectral cube was subdivided into numerous spatial patches (each of which is labeled according to the plant’s condition). This patch-wise approach greatly expands the number of training and testing samples, mitigating concerns about limited data quantity. Second, to ensure robustness, we employed plant-level cross-validation, so each fold’s test set always contained plants never seen during training. Regarding whether further increasing the dataset size would improve model accuracy: in general, deep learning methods benefit from larger and more diverse training sets. We do anticipate that adding more plants, potentially including different growth stages or environmental conditions, would further improve both the model’s performance and its capacity to generalize. Our current results, however, already indicate strong classification accuracy and the viability of the approach for early virus detection, even with this dataset size.

The patch size is another important parameter. We initially experimented with various patch sizes ranging from $8 \times 8$ to $32 \times 32$ in order to strike a balance between capturing the local spectral-spatial features critical for early disease detection and retaining a sufficient number of training samples for robust model learning. Very small patches (e.g., $8 \times 8$) sometimes caused confusion between healthy and diseased tissues, particularly around leaf veins or margins, potentially leading to overfitting. Conversely, using larger patches (e.g., $32 \times 32$) reduced the total number of patches available for training and increased the risk of blending healthy and infected regions within the same patch. Through iterative trials, we identified $16 \times 16$ as the most effective compromise: it is small enough to provide a large corpus of training samples per leaf, yet large enough to include meaningful context, such as subtle lesion patterns adjacent to healthy areas, crucial for accurate detection. We acknowledge that the optimal patch size may vary depending on factors like leaf morphology, disease progression stage, and sensor resolution; accordingly, future efforts could investigate multi-scale or adaptive patch extraction strategies to capture diverse phenotypic or pathological patterns within a single leaf.

Indubitably, this research has certain limitations. The proposed methodology ignores time since the temporal evolution of the plants’ reflectance is not taken into account. A future step towards incorporating time would be combining the 3D-CNN with the long short-term memory method, originally proposed by Hochreiter et al. [42]. Moreover, the hidden layers of neural networks act somewhat like a “black box”. This is an inherent drawback for the interpretation of the results of the proposed method. Finally, the proposed method was tested using hyperspectral imagery acquired in the controlled environment of a laboratory. Its accuracy in the real world (i.e., in field conditions or in greenhouses) may be different.

Further research is needed to address several key questions. Firstly, the optimal dimensions for square slices that capture the spatial patterns essential for disease detection must be determined. This can be achieved by comparing results across square slices of varying sizes using the same benchmark dataset. However, this approach would necessitate a larger dataset, as larger nonoverlapping square slices inherently reduce the number of available training samples. In this study, we selected nonoverlapping patches of size $16 \times 16$ through an iterative trial-and-error process as the smallest patches that yielded effective results. We recognize that the optimal patch size may vary based on factors such as leaf morphology, disease progression stage, and sensor resolution. Consequently, future efforts could explore multi-scale or adaptive patch extraction strategies to capture diverse phenotypic or pathological patterns within a single leaf. Additionally, in future studies, we plan to train on multi-genotype datasets to explore whether incorporating data from diverse plant backgrounds can further boost model robustness and early detection capabilities. Lastly, extending the proposed method to quantify the infection severity level (a task already performed for fungi [43]) could prove quite useful.

## Conclusions

The use of 3D-CNNs has been shown to be a reliable approach for early potexvirus-mediated disease detection in tobacco plants using hyperspectral images in the Vis/NIR spectral region (380-1023 nm). Instead of employing the most common, pixel-wise approach, a patch-wise approach has been adopted, and $16 \times 16 \times 61$ patches have been extracted from the hyperspectral images. The ERT algorithm has been employed for feature selection, yielding patches of dimensions $16 \times 16 \times 16$. These patches have been used as input samples for training and testing the 3D-CNN models. A 3D-CNN architecture has been proposed for tackling the 2-class problem of disease detection for each of the four viruses examined, achieving accuracies ranging from 0.78 to 0.87 when trained and tested on plants of genotype *AGO2*. At the plant level, the average overall accuracy has ranged from 0.68 to 0.89. The generalization capability of the model has also been assessed by testing it on hyperspectral images of plants of genotype *DCL2/4*, *NahG,* and wild-type, yielding accuracies from 0.48 to 0.75, from 0.57 to 0.83 and from 0.54 to 0.78 respectively. Summing up, the proposed model has attained high accuracies, only 6 DPI for PepMV SP13, PepMV PCH 06/104, and PVX, and at 8 DPI for BaMV, in agreement with plant systemic viral loads as detected by northern blot analysis, generalized well over different genotypes and outperformed the state-of-the-art methodologies it was benchmarked against.

## Data Availability

The datasets used and/or analyzed during the current study will be made available on a public repository upon paper publication.

## Abbreviations

AI: Artificial Intelligence
BaMV: Bamboo mosaic virus
BPNN: Back Propagation Neural Network
BRT: Boosted Regression Tree
CART: Classification and Regression Tree (CART)
CBSV: Cassava brown streak virus
CNN: Convolutional Neural Network
CP: coat protein
DPI: Days Post Inoculation
ELM: Extreme Learning Machine
ERT: Extremely Randomized Trees
GAN: Generative Adversarial Network
GLRaV: Grapevine leafroll-associated virus
GRBV: Grapevine red blotch-infected virus
GVCV: Grapevine vein clearing virus
LS-SVM: Least Squares Support Vector Machine
NIR: Near Infrared
PepMV: Pepino mosaic virus
PLS-DA: Partial Least-Squares Discriminant Analysis
PVX: Potato virus X
RF: Random Forest
SA: salicylic acid
SMV: Soybean mosaic virus
SPA: Successive Projections Algorithm
SVM: Support Vector Machine
TMV: Tobacco mosaic virus
TSWV: Tomato spotted wilt virus
TYLCV: Tomato yellow leaf curl virus
Vis/NIR: Visible/Near Infrared
WT: Wild Type
XGBoost: Extreme Gradient Boosting

## Footnotes

1 Repository will be made public upon paper acceptance.

## Code availability

The algorithmic implementation of the framework was developed in Python 3, using open-source Python libraries can be found at https://github.com/chadoulis/hyperspectralCube. The repository will publicly be released upon paper publication
